# PIWI-interacting RNA-36712 restrains breast cancer progression and chemoresistance by interaction with *SEPW1* pseudogene *SEPW1P* RNA

**DOI:** 10.1186/s12943-019-0940-3

**Published:** 2019-01-12

**Authors:** Liping Tan, Dongmei Mai, Bailin Zhang, Xiaobing Jiang, Jialiang Zhang, Ruihong Bai, Ying Ye, Mei Li, Ling Pan, Jiachun Su, Yanfen Zheng, Zexian Liu, Zhixiang Zuo, Qi Zhao, Xiaoxing Li, Xudong Huang, Jie Yang, Wen Tan, Jian Zheng, Dongxin Lin

**Affiliations:** 1 State Key Laboratory of Oncology in South China and Collaborative Innovation Center for Cancer Medicine, Sun Yat-sen University Cancer Center, Guangzhou, China; 2 Department of Breast Surgery, National Cancer Center/Cancer Hospital, Chinese Academy of Medical Sciences and Peking Union Medical College, Beijing, China; 3 Department of Surgical Oncology, Sun Yat-sen University Cancer Center, Guangzhou, China; 4 Department of Pathology, Sun Yat-sen University Cancer Center, Guangzhou, China; 5 Department of Etiology and Carcinogenesis, National Cancer Center/Cancer Hospital, Chinese Academy of Medical Sciences and Peking Union Medical College, Beijing, China

**Keywords:** piRNA, Breast cancer, P53, Drug sensitivity, SEPW1

## Abstract

**Background:**

Breast cancer is one of the most common malignancies and the major cause of cancer-related death in women. Although the importance of PIWI-interacting RNAs (piRNAs) in cancer has been increasingly recognized, few studies have been explored the functional mechanism of piRNAs in breast cancer development and progression.

**Methods:**

We examined the top 20 highly expressed piRNAs based on the analysis of TCGA breast cancer data in two patient cohorts to test the roles of piRNAs in breast cancer. The effects of piRNA-36,712 on the malignant phenotypes and chemosensitivity of breast cancer cells were detected in vitro and in vivo. MS2-RIP and reporter gene assays were conducted to identify the interaction and regulation among piRNA-36,712, miRNAs and *SEPW1P*. Kaplan-Meier estimate with log-rank test was used to compare patient survival by different piRNA-36,712 expression levels.

**Results:**

We found piRNA-36,712 level was significantly lower in breast cancer than in normal breast tissues and low level was correlated with poor clinical outcome in patients. Functional studies demonstrated that piRNA-36,712 interacts with RNAs produced by *SEPW1P*, a retroprocessed pseudogene of *SEPW1*, and subsequently inhibits SEPW1 expression through competition of *SEPW1* mRNA with *SEPW1P* RNA for microRNA-7 and microRNA-324. We also found that higher SEPW1 expression due to downregulation of piRNA-36,712 in breast cancer may suppress P53, leading to the upregulated Slug but decreased P21 and E-cadherin levels, thus promoting cancer cell proliferation, invasion and migration. Furthermore, we found that piRNA-36,712 had synergistic anticancer effects with the paclitaxel and doxorubicin, two chemotherapeutic agents for breast cancer.

**Conclusions:**

These findings suggest that piRNA-36,712 is a novel tumor suppressor and may serve as a potential predictor for the prognosis of breast cancer patients.

**Electronic supplementary material:**

The online version of this article (10.1186/s12943-019-0940-3) contains supplementary material, which is available to authorized users.

## Background

In last decade, non-coding RNAs (ncRNAs) such as microRNAs and long ncRNA (lncRNA) have attracted considerable attention in cancer researches because of their roles in regulating gene expression and functionally interacting with other molecules. Recently, another subclass of ncRNAs, PIWI-interacting RNAs (piRNAs), has been thought to be emerging players in cancer genomics [1]. piRNAs are small ncRNAs consisting of 24–32 nucleotides and specifically interact with PIWI proteins that are members of Argonaute protein family. Some piRNAs were initially identified in the germline [2], where they play important functions such as repression of transposable elements and epigenetic regulations of gene expression [1, 3–5]. It has been shown that piRNAs are not only expressed in the germline but also occur and function in human somatic tissues with tissue expression specificity, despite the lower number of expressed piRNAs in somatic tissues than that in the germline [6, 7]. With the advance in the field of next generation sequencing, the roles of piRNAs in human diseases including cancer have become interesting [8] and several studies have reported the aberrant expressions of piRNAs in some types of human cancer [7, 9, 10]. Furthermore, a growing body of evidence has indicated that aberrant expression of certain piRNAs may participate in tumorigenesis and are also relevant to the prognosis of cancer [8, 11–13]. Although the importance of piRNAs in cancer has been increasingly recognized, little has been known about piRNAs in this field as compared with microRNAs that are far less abundant than piRNAs but have been implicated in almost every cancer type [6]. In addition, few studies have been explored the functional mechanism of piRNAs in cancer development and progression [12–17]. Therefore, it would be interesting and significant to identify cancer-related piRNAs and elucidate their acting mechanisms.

Breast cancer is one of the most common malignancies and the major cause of cancer-related death in women [18, 19]. Genomic instability especially mutations of BRCA1 and BRCA2 confer high risks of breast cancer [20–22]. Breast cancer is a heterogeneous disease exhibiting a range of biologic and clinical behaviors. In clinical, breast cancer had been divided into five subtypes according to the expression patterns of estrogen receptor (ER), progesterone receptor (PR) and human epidermal growth factor receptor 2 (HER2), which was useful in predicting clinical outcome and selecting appropriate therapy [23]. ER/PR positive confer sensitivity to endocrinotherapy, while HER2 amplification leading to a good response to HER2 antagonists. And negative for ER, PR and HER2 were related to poor prognosis [22, 24]. Although great advances have been achieved, the molecular mechanism for breast cancer pathogenesis and progression is still not fully elucidated, impeding precise and personalized treatment. Besides molecular subtypes of breast cancer, several criteria including gene-expression signature have been used for predicting clinical outcome and chemosensitivity [25–28], however, not all patients are benefited from treatment guided by the prediction systems and therefore have worse outcomes [29, 30], suggesting that other genomic factors and mechanism should be explored for better explanation of the cancer. Since piRNAs are emerging to play important roles in cancer, we hypothesized that some of piRNAs might be implicated in the development and progression of breast cancer. To examine this hypothesis, we systematically analyzed the expression profile of piRNAs in breast cancer using whole-genome sequencing data from The Cancer Genome Atlas (TCGA) and then validated in our clinical breast cancer tissue samples.

Here, we report the identification of piRNA-36,712 (piR-36,712) that is significantly low expressed in breast cancer compared with non-tumor tissues and acts as a possible tumor suppressor. We have revealed a complex molecular mechanism for the function of piR-36,712 in breast cancer cells. Furthermore, we have also found that upregulated expression of piRNA-36,712 has synergistic anticancer effect with chemotherapeutic agents on breast cancer cells.

## Methods

### Study subjects

Samples were collected from individuals clinically defined breast cancer at Sun Yat-sen University Cancer Center (SYSUCC, Guangzhou) or Cancer Hospital, Chinese Academy of Medical Science (CHCAMS, Beijing) under the approval of ethical committee. Detailed information for the involved samples of this study is provided in Additional file 1: Materials and Methods.

### Public data mining

piRNA selection, somatic copy number alterations (SCNAs) and DNA methylation status of piR-36,712 gene were performed based on the data from TCGA and GEO database under a series of independent bioinformatic analyses. RNA-miRNA binding interactions were analyzed by publicly available algorithms. See Additional file 1: Materials and Methods for details.

### Cell lines and cell culture

All cell lines in this study were purchased from the Cell Bank of Type Culture Collection of the Chinese Academy of Sciences, Shanghai Institute of Biochemistry and Cell Biology. Cell culture and all cellular assays are described in Additional file 1: Materials and Methods.

### Immunofluorescence assays

Cells (1.5 × 10^3^~1. 5 × 10^4^ per well) were seeded in 96-well plates. Cells were labeled with 30 μmol/L 5-Ethynyl-2′- deoxyuridine (EdU, RIBOBIO) for 2 h, stained with 0.4% paraformaldehyde for 30 min, stained with apollo reaction and photoed. The flow cytometry assay for testing EdU staining was done following the manufacturer’s instructions.

### Animal experiments

All the experiments of xenograft tumor were conducted in accordance with relevant institutional and national guidelines and regulations. These animal experimental procedures were given in Additional file 1: Materials and Methods.

### Lentivirus production and vector construction

Vectors expressing piR-36,712 or its antisense, wild type or mutant SEPW1 and SEPW1P as well as MS2-12X system were constructed and details were provided in Additional file 1: Materials and Methods.

### Real-time PCR analysis, reporter assays and pharmacological detection

Intensive description was provided in Additional file 1: Materials and Methods and Additional file 8: Table S5.

### Northern blot, immunoblotting and immunoprecipitation

Details were given in Additional file 1: Materials and Methods and Additional file 9: Table S6.

### Statistical analysis

Kaplan-Meier estimate with log-rank test was used to compare patient survival by different piR-36,712 expression levels. Cox proportional hazards models were used to identify independent significant variables. Hazard ratios (HRs) and 95% confidence intervals (CIs) were calculated with age, menopausal status, ER status, PR status, HER2 status, Ki67 index, histological grade, TNM stage, number of positive axillary lymph node and adjuvant chemotherapy as covariates. For functional analysis, results were presented as mean ± SEM. Comparison of mean between two groups was conducted using Student’s *t*-test, while the comparison for more than two groups was conducted using one-way ANOVA. Data in abnormal distribution were analyzed by non-parametric test. All statistical analyses were performed using SPSS 20.0 (IBM, US) and *P* < 0.05 was considered significant.

## Results

### piR-36,712 is downregulated in breast cancer and associated with clinical outcomes

**Fig. 1:**
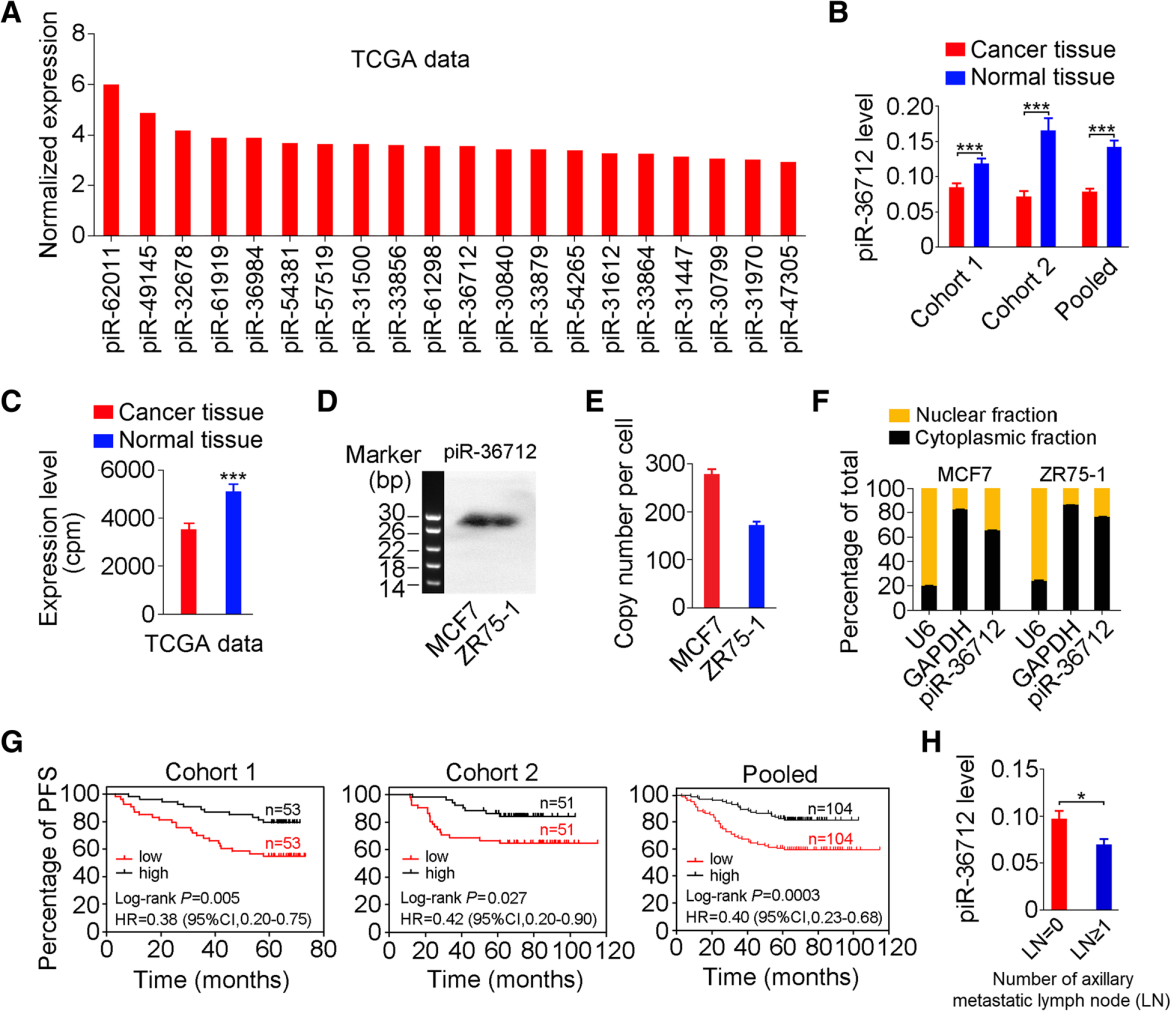
piR-36,712 is downregulated in breast cancer and correlated with clinical outcomes in patients. **a** The top 20 highly expressed piRNAs in breast cancer based on the analysis of TCGA data. **b** The expression levels of piR-36,712 in breast cancer and paired non-tumor tissues from patients recruited at SYSUCC (Cohort 1) and CHCAMS (Cohort 2). Data are mean ± SEM, ***, *P* < 0.001). **c** The expression levels of piR-36,712 in 103 paired breast cancer and non-tumor tissues from TCGA database (mean ± SEM; ***, *P* < 0.001). **d** Northern blot of piR-36,712 in breast cancer cells. **e** piR-36,712 copy number per cell in MCF7 and ZR75–1 cell lines (mean ± SEM). **f** Distribution of piR-36,712 in cytoplasm and nucleus of breast cancer cells with U6 or GAPDH as nucleus or cytoplasm markers (% ± SEM). **g** Kaplan-Meier estimates of progression-free survival time in breast cancer patients at SYSUCC (Cohort 1) and CHCAMS (Cohort 2) stratified by piR-36,712 levels in tumor. HR, hazard ratio; CI, confidence interval. **h** The expression levels of piR-36,712 in breast cancer with (LN ≥ 1) or without (LN = 0) axillary lymph node (LN) metastasis (mean ± SEM; *, *P* < 0.05)

We firstly analyzed the expression profile of piRNAs in breast cancer using TCGA data and then performed qRT-PCR to examine the levels of the top 20 highly expressed piRNAs (Fig. 1a) in a set of 106 breast cancer and matched normal tissue samples collected from Sun Yat-sen University Cancer Center (SYSUCC, Guangzhou). We observed a significant downregulation of piR-36,712 in breast cancers compared with their nontumor tissues (Fig. 1b); but the expression levels of other 19 piRNAs were not significantly different (Additional file 2: Figure S1A). The results were validated in another set of paired breast cancer and normal tissue samples (*N* = 102) obtained from Cancer Hospital, Chinese Academy of Medical Sciences (CHCAMS, Beijing) (Fig. 1b). Additional analysis of piR-36,712 levels in 103 paired breast cancer and non-tumor counterparts in the TCGA database also confirmed substantially lower expression of this piRNA in breast cancer (Fig. 1c). Analysis of piR-36,712 gene alterations in breast cancer of the TCGA database showed significant hypermethylation (Additional file 2: Figure S1B and S1C) and deletion (Additional file 2: Figure S1D). We confirmed the existence of piR-36,712 in breast cancer cell lines MCF7 and ZR75–1 by Northern blotting (Fig. 1d) and the absolute copy number of this piRNA was 160 to 300 copies per cell (Fig. 1e). The cellular distribution analysis showed that the major amount of piR-36,712 is in cytoplasm (Fig. 1f).

We then examined the associations of piR-36,712 levels in breast cancer tissues and clinical outcomes in our breast cancer patient cohorts (Additional file 3: Table S1 and Additional file 4: Table S2) and found that individuals with higher level of piR-36,712 had significantly longer progression-free survival (PFS) in both SYSUCC and CHCAMS patient cohorts and combined sample (Fig. 1g). We also observed a substantial downregulation of piR-36,712 in breast cancer with axillary lymph node metastasis compared with that without lymphatic metastasis (Fig. 1h). Multivariate COX regression analysis with age, menopausal status, ER status, PR status, HER2 status, Ki67 index, histological grade, number of positive node, TNM stage and adjuvant chemotherapy as covariates showed that piR-36,712 level was an independent prognostic factor for PFS, with a hazard ratio being 0.39 (95% CI = 0.23–0.67; Additional file 5: Table S3). We did not find any correlation between piR-36,712 level in breast cancer and other clinical parameters except for the number of lymph nodes (Additional file 6: Table S4).

### piR-36,712 suppresses malignant phenotypes of breast cancer cells

**Fig. 2:**
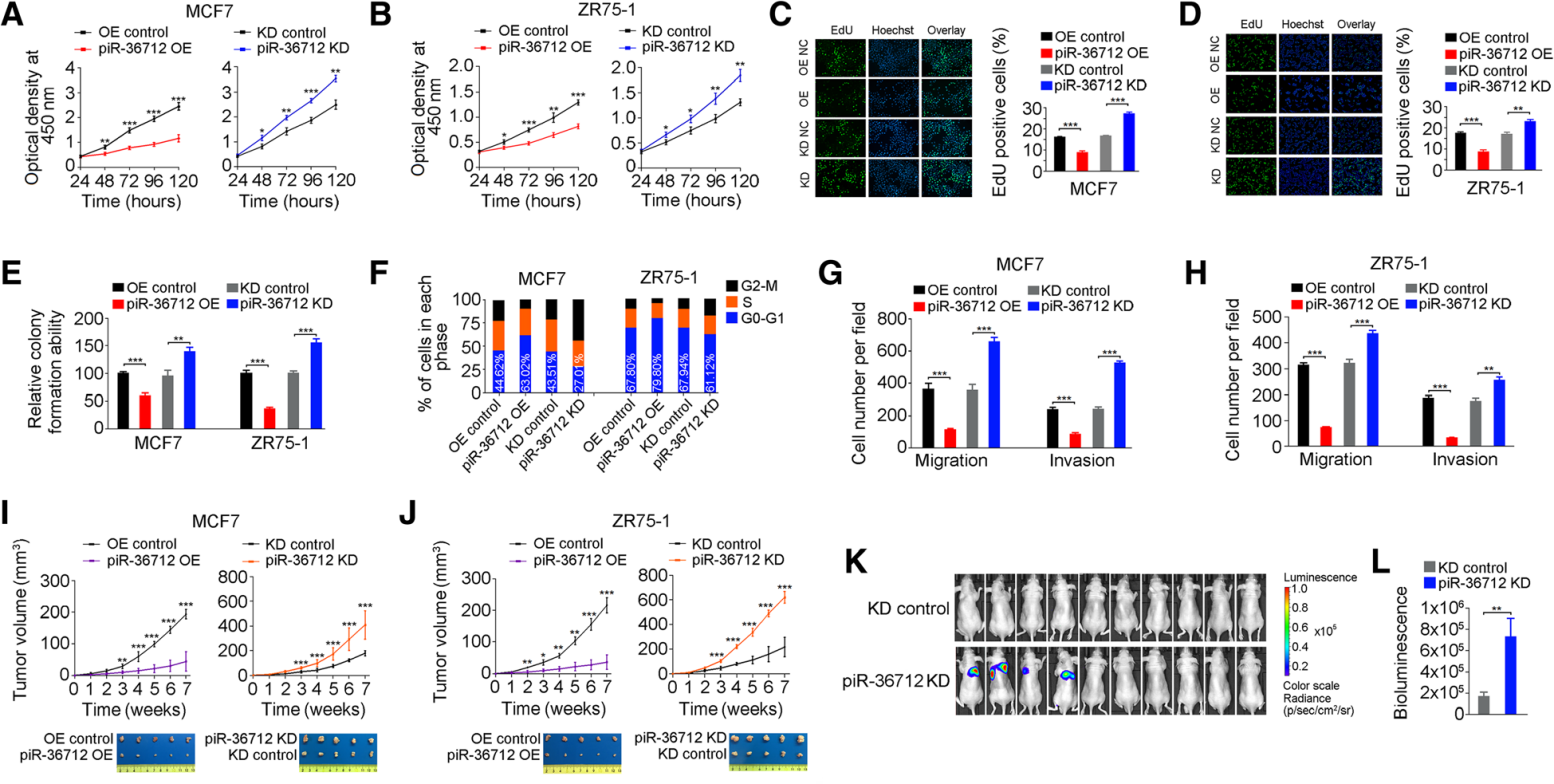
piR-36,712 suppresses malignant phenotypes of breast cancer cells. **a**, **b** Effect of piR-36,712 expression on MCF7 and ZR75–1 cell proliferation tested by CCK8 assay (mean ± SEM; *, *P* < 0.05; **, *P* < 0.01 and ***, *P* < 0.001). **c**, **d** The fluorescent thymidine analog EdU was used to identify proliferative cells by labeling their DNA (green signal). Nuclei labeled with hoechst are in blue. Representative images (left) and quantitative statistics by flow cytometry (right) (mean ± SEM, *n* = 3; NS, **, *P* < 0.01 and ***, *P* < 0.001). **e** Effect of piR-36,712 expression on colony formation ability of breast cancer cells. Results present colony formation ability relative to control (mean ± SEM; **, *P* < 0.01 and ***, *P* < 0.001). **f** Effect of piR-36,712 overexpression or knockdown on MCF7 and ZR75–1 cell cycle progression. **g**, **h** Effects of piR-36,712 on the abilities of MCF7 and ZR75–1 cell migration and invasion (means ± SEM; **, *P* < 0.01; ***, *P* < 0.001). **i**, **j** Effects of piR-36,712 expression on the growth of MCF7 and ZR75–1 xenografts in mice (mean ± SEM, *N* = 5 in each group; **, *P* < 0.01 and ***, *P* < 0.001). **k**, **l** Effects of piR-36,712 expression on MCF7 cell metastasis in mice. Luminescence imaging of metastases (**k**) and quantification of radiance intensity (**l**). Data are mean ± SEM, *N* = 10 in each group; ***, *P* < 0.001

Since piR-36,712 is substantially downregulated in breast cancer, we wanted to know whether it has effect on breast cancer cell phenotypes. We found that overexpression of piR-36,712 significantly suppressed MCF7 and ZR75–1 proliferation; whereas knockdown of piR-36,712 significantly promoted cell proliferation from the CCK8 and EdU incorporation assays (Additional file 2: Figure S2A and Fig. 2a-d). The similar results were also seen in colony formation ability assays with the same MCF7 and ZR75–1 cells (Fig. 2e and Additional file 2: Figure S2B). Overexpression of piR-36,712 led to accumulation of cancer cells in G0/G1 phase suggesting a cell cycle arrest; however, knockdown of piR-36,712 significantly decreased the number of cancer cells in G0/G1 phase but increased the number of cancer cells in G2 phase (Fig. 2f and Additional file 2: Figure S2C). However, neither overexpression nor knockdown of piR-36,712 had a measurable effect on the apoptosis of these two cell lines (Additional file 2: Figure S2D and S2E). We then examined whether such function of cell cycle arrest has impact on cancer cell growth in vivo and found that the growth rates of MCF7 and ZR75–1 xenografts overexpressing piR-36,712 were significantly slower than those of control counterparts (Fig. 2i and j). In contrast, the growth rates of xenografts with piR-36,712 knockdown were greatly faster than those of controls (Fig. 2i and j). We also found that overexpression of piR-36,712 significantly suppressed MCF7 and ZR75–1 cell migration and invasion in vitro, but knockdown of piR-36,712 in the same cells substantially enhanced these malignant phenotypes (Fig. 2g and h, Additional file 2: Figure S2F and S2G)**.** In vivo assays also demonstrated this role of piR-36,712. We found that when injected into mice tail-vein, MCF7 cells with piR-36,712 knockdown formed lung metastatic cancer in 40% (4/10) animals in a period of 8 weeks after injection; however, injection of the same cells without piR-36,712 knockdown formed almost no lung metastases (Fig. 2k and l, Additional file 2: Figure S2H and S2I).

### piR-36,712 directly interacts with *SEPW1P* RNA in breast cancer cells

**Fig. 3:**
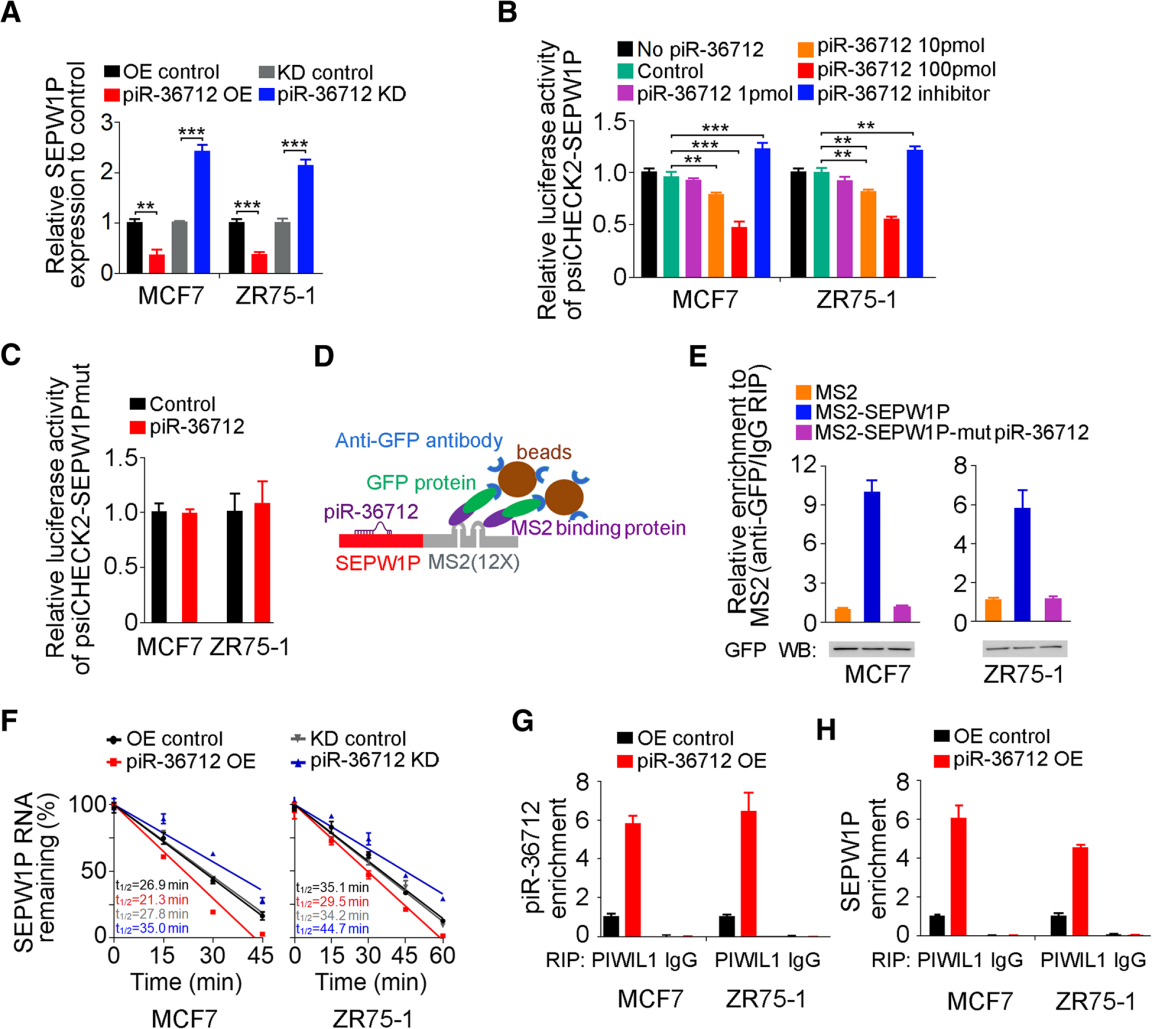
piR-36,712 interacts with *SEPW1P* RNA. **a** Levels of *SEPW1P* RNA in MCF7 and ZR75–1 cells with stable overexpression (OE) or knockdown (KD) of piR-36,712 (mean ± SEM; **, *P* < 0.001; ***, *P* < 0.0001). **b** Relative reporter gene activity of psiCHECK2 vector bearing *SEPW1P* in MCF7 and ZR75–1 cells co-transfected with indicated amount of piR-36,712 mimic or inhibitor (mean ± SEM; **, *P* < 0.001; ***, *P* < 0.0001). **c** Relative reporter gene activity of psiCHECK2 vector bearing *SEPW1P* with mutation at putative binding site of piR-36,712 in MCF7 and ZR75–1 cells co-transfected with 100 pmol of piR-36,712 mimic. **d** Schematic diagram of MS2-RNA immunoprecipitation (RIP) assay. **e** MS2-RIP and qRT-PCR analysis shows interaction of piR-36,712 with *SEPW1P* RNA in MCF7 and ZR75–1 cells. **f** Decay of *SEPW1P* RNA in MCF7 and ZR75–1 cells with piR-36,712 overexpression or knockdown treated with actinomycin D determined by qRT-PCR. **g, h** RIP and qRT-PCR analysis shows significant increased association with PIWIL1 protein of piR-36,712 (**g**) and *SEPW1P* RNA (**h**) in MCF7 and ZR75–1 cells

To shed light on the mechanism underlying the piR-36,712 actions, we performed several assays. Firstly, we examined whether piR-36,712 has the *cis* regulation effect on genes located at or near the same genomic locus by determining the RNA levels of 7 genes (*NKAIN1*, *RPL21P22*, *PUM1*, *SDC3*, *LAPTM5*, *MATN1* and *SEPW1P*) within about 0.3 M-bases centering the gene producing piR-36,712 in cells with or without piR-36,712 knockdown (Additional file 2: Figure S3A). We found no significant expression changes of these genes except for *SEPW1P* RNA, which was significantly upregulated when piR-36,712 was knocked down (Fig. 3a and Additional file 2: Figure S3B). However, the elevated level of *SEPW1P* RNA resulted from knockdown of piR-36,712 seems not caused by the *cis* regulation because further in silico analysis revealed a putative piR-36,712-binding site (from 62 to 95 nucleotides) at *SEPW1P* RNA (Additional file 2: Figure S3C), suggesting a direct interaction between piR-36,712 and *SEPW1P* RNA. Indeed, reporter gene assays with psiCHECK2 bearing full length of *SEPW1P* cDNA (psiCHECK2-SEPW1P) or full length of *SEPW1P* cDNA with mutations at the putative piR-36,712-binding site (psiCHECK2-SEPW1Pmut) indicated the possible interaction between these two RNAs (Fig. 3b and c, Additional file 2: Figure S3D). We then performed MS2-based RNA immunoprecipitation (RIP) assays in cells in natural state (Fig. 3d) and quantitative PCR analysis and found that piR-36,712 was greatly enriched in MS2-SEPW1P compared with empty MS2 or MS2-SEPW1P-mut (Fig. 3e). We also found that overexpression of piR-36,712 in breast cancer cells significantly reduced *SEPW1P* RNA levels (Fig. 3a). The RNA decay assays found that overexpression of piR-36,712 reduced the stability of *SEPW1P* RNA but knockdown of piR-36,712 increased the stability (Fig. 3f). Previous study has revealed in species of mouse that, piRNA interacting with MIWI protein can form RNA-induced silencing complex (RISC), then mediating the cleavage of piRNA-targeted mRNA [31]. The analysis of RIP with PIWIL1 antibody, the human PIWI protein highly homologous to MIWI [32, 33], followed by RT-qPCR also revealed a remarkably increase in recruitment of *SEPW1P* RNA to the piR-36,712/PIWIL1 complex in cells overexpressing piR-36,712 (Fig. 3g and h). These results jointly provided strong evidence that piR-36,712 may directly interact with *SEPW1P* RNA.

### piR-36,712 inhibits SEPW1 expression by interacting with *SEPW1P* RNA

**Fig. 4:**
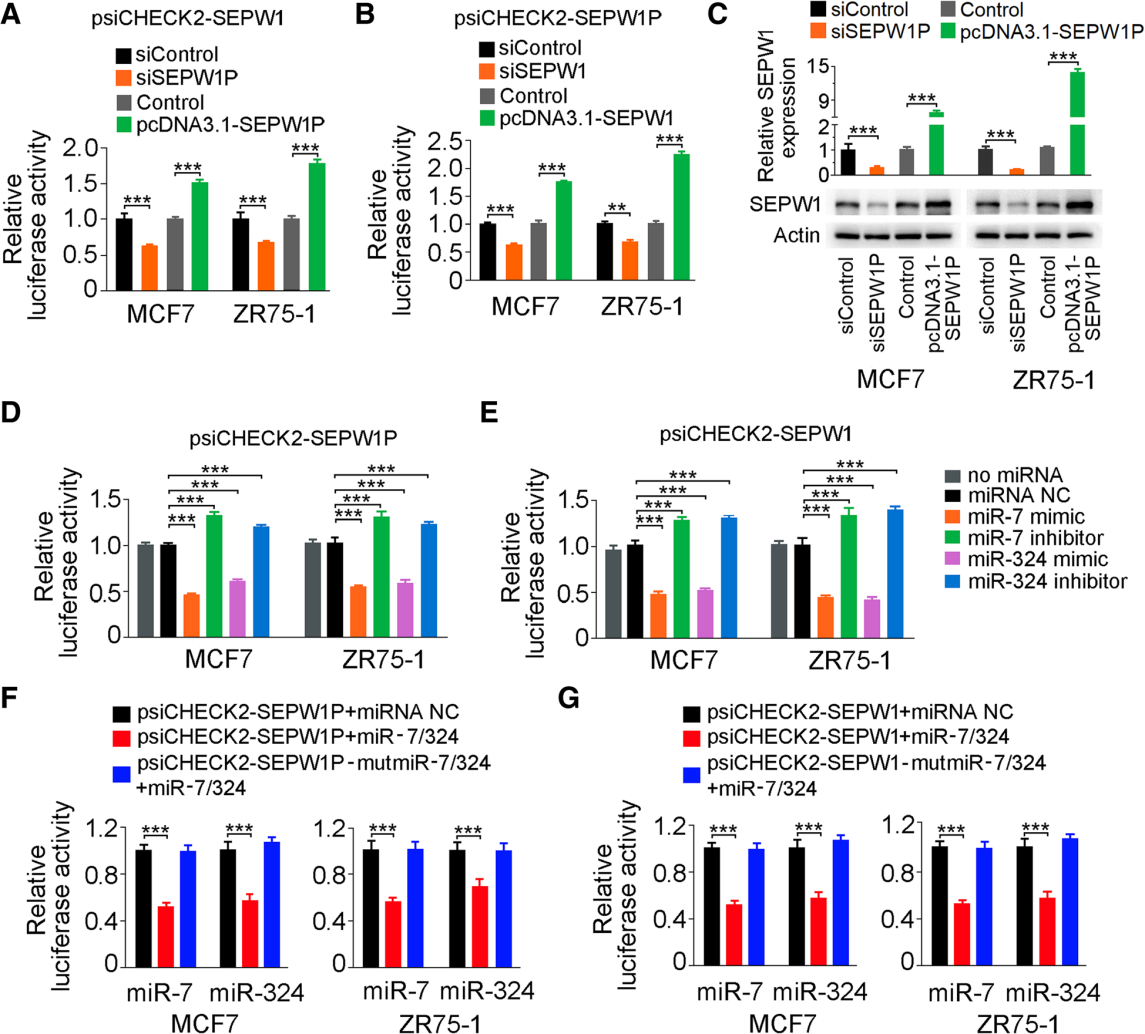
Competition of *SEPW1P* RNA with *SEPW1* mRNA for miR-7 and miR-324. **a** Relative luciferase activity of psiCHECK2 vector bearing *SEPW1* 3’UTR in MCF7 and ZR75–1 cells with overexpression or knockdown of *SEPW1P* by cotransfected with pcDNA3.1-SEPW1P or siSEPW1P. **b** Relative reporter gene activity of psiCHECK2 vector bearing *SEPW1P* in MCF7 and ZR75–1 cells with overexpression or knockdown of *SEPW1* by cotransfected with pcDNA3.1-SEPW1 or -siSEPW1. **c** Levels of *SEPW1* mRNA and protein in MCF7 and ZR75–1 cells with overexpression (pcDNA3.1-SEPW1P) or knockdown (siSEPW1P) of SEPW1. **d** Relative reporter gene activity of constructs bearing *SEPW1P* (**d**) or *SEPW1* 3’UTR (**e**) in MCF7 and ZR75–1 cells cotransfected with miR-7 or miR-324 mimic or inhibitor. **f**, **g** Relative reporter gene activity of psiCHECK2 vector bearing *SEPW1P* (**f**) or *SEPW1* 3’UTR (**g**) with mutations at putative binding site of miR-7 or miR-324 in MCF7 and ZR75–1 cells cotransfected with miR-7 or miR-324 mimic or inhibitor. Shown are mean ± SEM; **, *P* < 0.001; ***, *P* < 0.0001

We next wanted to know why the interaction of piR-36,712 with *SEPW1P* RNA is able to suppress the malignant phenotypes of breast cancer cells. Since *SEPW1P* is a pseudogene that might function as a long non-coding RNA to regulate its protein-coding counterpart (*SEPW1*) via a mechanism known as competitive endogenous RNA (ceRNA) [34–37], we firstly examined the regulatory relationship between *SEPW1P* and *SEPW1* using reciprocal reporter gene assays and found that the luciferase activity produced by psiCHECK2-SEPW1 was significantly reduced when *SEPW1P* was knocked down in cells but significantly increased when *SEPW1P* expression was overexpressed (Fig. 4a). Similarly, the luciferase activity produced by psiCHECK2-SEPW1P was significantly decreased when *SEPW1* was knocked down in cells but significantly increased when *SEPW1* was overexpressed in cells (Fig. 4b). Furthermore, the RNA and protein levels of SEPW1 were downregulated when *SEPW1P* was suppressed by its siRNA, while upregulated when *SEPW1P* was overexpressed (Fig. 4c). These results indicated that *SEPW1* expression level may be regulated by *SEPW1P* expression.

**Fig. 5:**
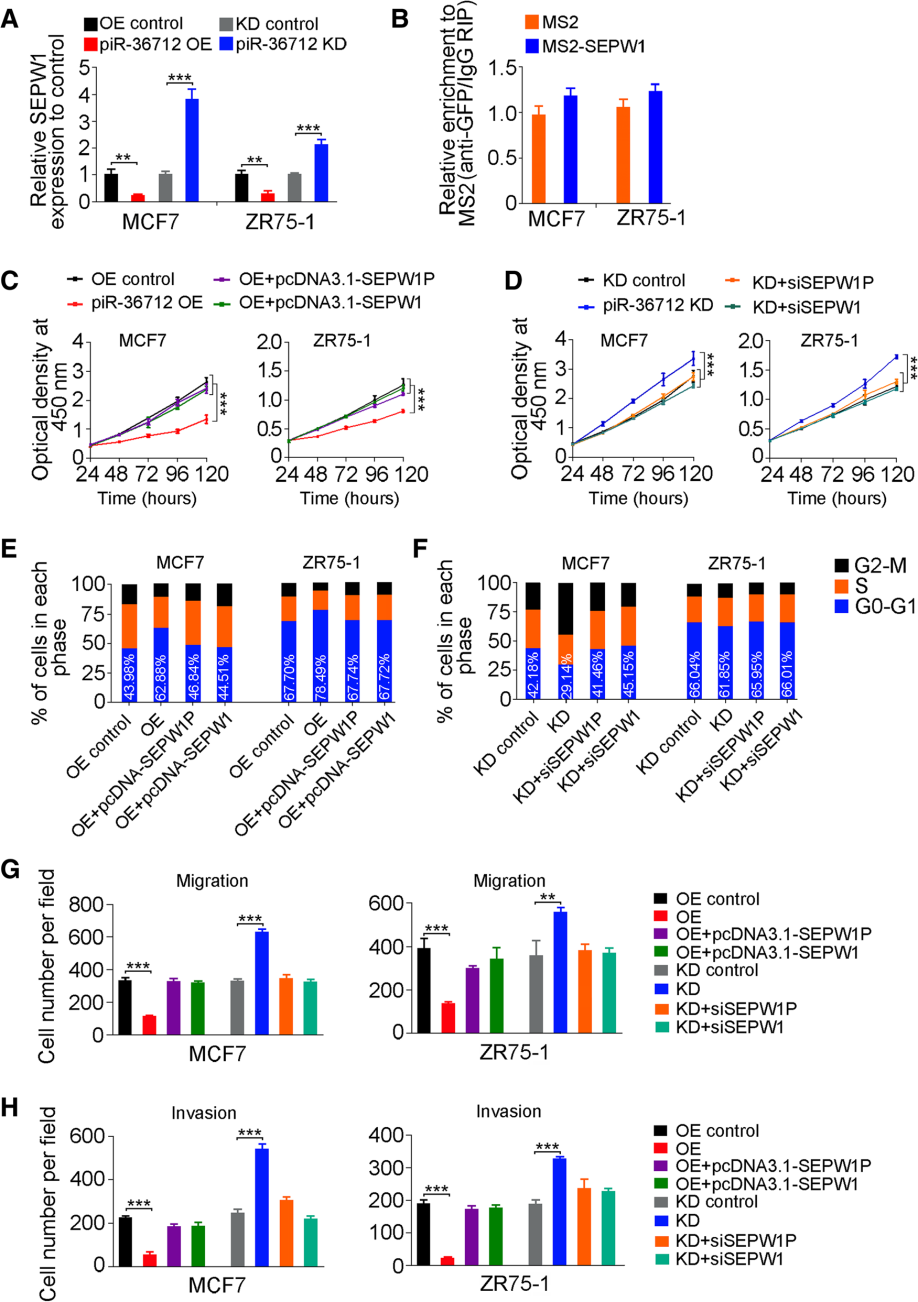
piR-36,712 inhibits *SEPW1* expression and functions by interaction with *SEPW1P* RNA. **a** Levels of *SEPW1* mRNA in MCF7 and ZR75–1 cells with stable overexpression (OE) or knockdown (KD) of piR-36,712 (mean ± SEM; **, *P* < 0.001; ***, *P* < 0.0001). **b** MS2-RIP and qRT-PCR analysis shows interaction of piR-36,712 with *SEPW1* mRNA. **c**, **d** Effects of OE or KD of *SEPW1P* or *SEPW1* on breast cancer cell proliferation induced by OE or KD of piR-36,712 (mean ± SEM; ***, *P* < 0.001). **e**, **f** Effects of OE or KD of *SEPW1P* or *SEPW1* on breast cancer cell cycle progression induced by OE or KD of piR-36,712. **g**, **h** Effects of OE or KD of *SEPW1P* or *SEPW1* on breast cancer cell migration and invasion induced by OE or KD of piR-36,712 (mean ± SEM; *, *P* < 0.05; **, *P* < 0.01 and ***, *P* < 0.001)

Because miRNAs play a critical role in regulating gene expression, especially through the ceRNA mechanism, we then sought to identify miRNAs that may target both *SEPW1* and *SEPW1P* RNAs. In silico analysis with 4 publicly available algorithms suggested 5 miRNAs (miR-7, miR-216, miR-324, miR-422 and miR-641) that might have the interaction effect and were selected for further examination (Additional file 2: Figure S4A and Additional file 7: Extended Data Sheet 1). We first examined the abundance of these miRNAs in MCF7 and ZR75–1 cells and found that only miR-7 and miR-324 were relatively abundant (150 and 190 copies per cell, respectively) but the others were extremely low (Additional file 2: Figure S4B), which were excluded from the further investigations. Reporter gene assays showed that overexpression of miR-7 or miR-324 were able to repress the luciferase activity produced by psiCHECK2-SEPW1P or psiCHECK2-SEPW1, while knockdown of miR-7 or miR-324 increased the luciferase activities produced by the reporter plasmids (Fig. 4d and e). Reporter assays with mutated 3’UTR of *SEPW1* or *SEPW1P* (Additional file 2: Figure S4C and S4D) showed diminished effect of miR-7 or miR-324 (Fig. 4f and g). These results indicated that *SEPW1P* RNA may compete with *SEPW1* RNA for miR-7 and miR-324. Since *SEPW1P* and *SEPW1* RNA are high homologues in their sequences, we therefore examined whether piR-36,712 may also interact with *SEPW1* RNA as with *SEPW1P* RNA. We found that despite a significant change of *SEPW1* mRNA level in cells with overexpression or knockdown of piR-36,712 (Fig. 5a), MS2-based RIP assays did not support direct interaction between piR-36,712 and *SEPW1* mRNA (Fig. 5b). Sequence analysis also indicated that piR-36,712 may not bind to *SEPW1* RNA as it binds to *SEPW1P* RNA (Additional file 2: Figure S3C). These results indicated that suppression of malignant phenotypes of breast cancer cells by piR-36,712 may be mediated by *SEPW1P* and *SEPW1*. Indeed, overexpression of *SEPW1P* or *SEPW1* could reverse the inhibitory effect of piR-36,712 on proliferation of MCF7 and ZR75–1 cells (Fig. 5c) and silencing *SEPW1P* or *SEPW1* expression abolished the enhancement of MCF7 and ZR75–1 proliferation caused by knockdown of piR-36,712 (Fig. 5d). Similar rescue effects of *SEPW1P* or *SEPW1* were seen in assays examining cell cycle process (Fig. 5e and f, Additional file 2: Figure S5A and S5B) and migration and invasion abilities (Fig. 5g and h, Additional file 2: Figure S5C-F) of breast cancer cells**.** Together, these results suggested that the interaction of piR-36,712 with *SEPW1P* RNA may promote miR-7 and miR-324 to target *SEPW1* RNA.

### piR-36,712 functions as tumor suppressor via upregulation of P53

**Fig. 6:**
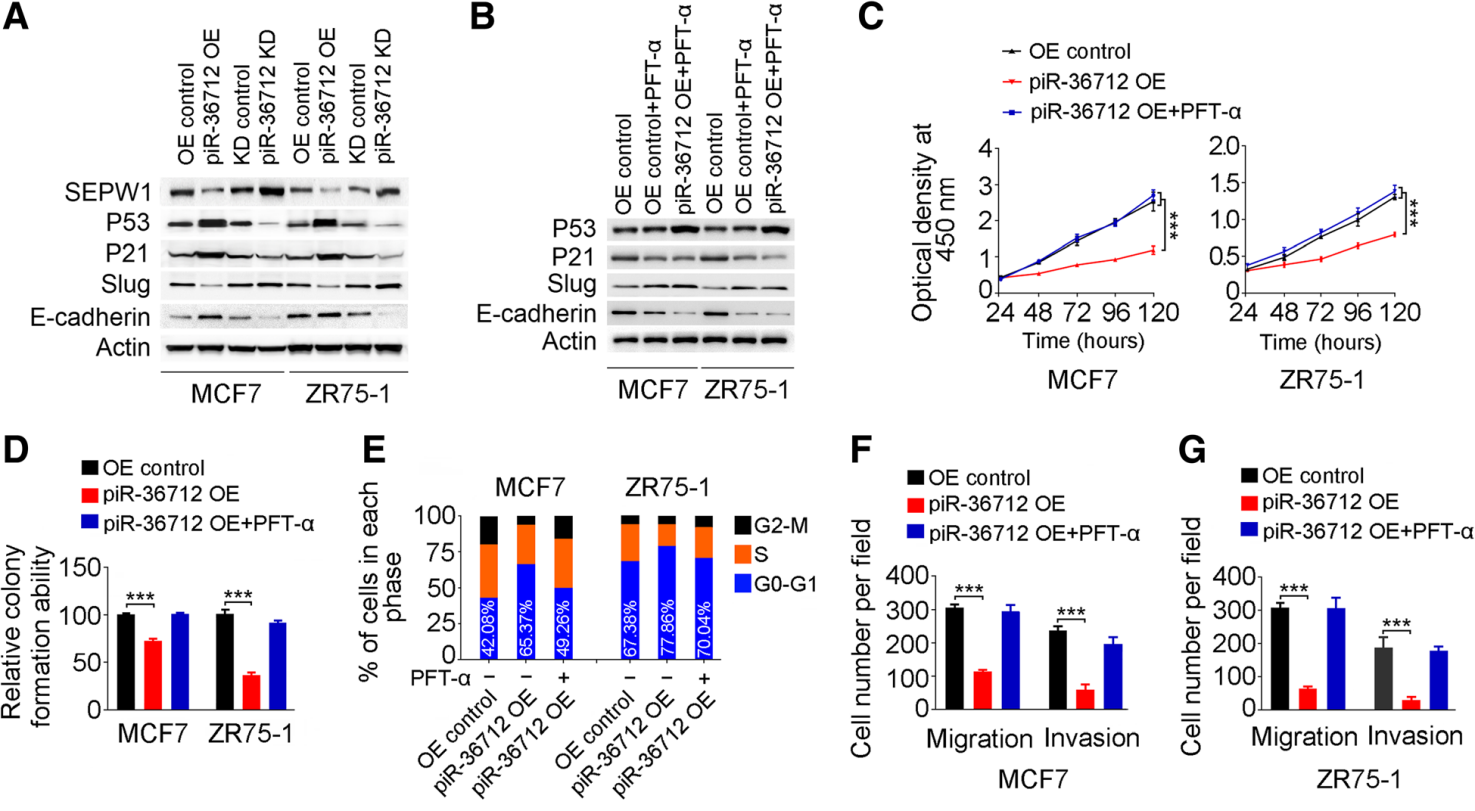
piR-36,712 suppresses the malignant phenotypes of breast cancer cells via P53. **a** Effects of piR-36,712 on expressions of SEPW1 and its downstream P53, P21, Slug and E-cadherin in MCF7 and ZR75–1 cells. **b** Inhibitory effect of PFT-α on expression of P53 and its downstream in MCF7 and ZR75–1 cells overexpressing piR-36,712. **c** Suppression of P53 by PFT-α inhibits the inhibitory effect of piR-36,712 on proliferation of MCF7 and ZR75–1 cells. **d** Suppression of P53 by PFT-α suppresses the inhibitory effect of piR-36,712 on colony formation ability of MCF7 and ZR75–1 cells. **e** Suppression of P53 by PFT-α inhibits the effect of piR-36,712 on G1 phase cell cycle arrest in MCF7 and ZR75–1 cells. **f** Suppression of P53 by PFT-α inhibits the inhibitory effect of piR-36,712 on MCF7 and ZR75–1 cell migration and invasion

Previous studies showed that depletion of SEPW1 increased P53 and P21 activities by depressing their degradation via ubiquitination, resulting in G1 cell cycle arrest [36, 37]. We therefore analyzed whether piR-36,712 may enhance P53 and P21 activities by suppressing SEPW1. Indeed, we found that overexpression of piR-36,712 in breast cancer cells considerably decreased SEPW1 level but increased P53 and P21 levels; however, knockdown of piR-36,712 substantially increased SEPW1 levels but decreased P53 and P21 levels (Fig. 6a). We also found that upregulation of P53 by piR-36,712 substantially reduced SLUG but increased E-CADHERIN level (Fig. 6a), which is consistent with previous reports suggesting that P53 may suppress cancer invasion by inducing MDM2-mediated degradation of SLUG, a transcription repressor that regulates expression of E-CADHERIN [38, 39]. To further verify that the tumor suppressor function of piR-36,712 is finally mediated by P53, we used PFT-α, a P53 pharmacological inhibitor, to explore whether it has any effects on the malignant phenotypes of breast cancer cells caused by piR-36,712. Western blot showed that MCF7 and ZR75–1 cells treated with PFT-α at a concentration of 10 μM for 48 h had a substantially decreased P21 level (Fig. 6b) and suppressed cell proliferation by piR-36,712 overexpression was considerably diminished when cells were treated with PFT-α (Fig. 6c and d, Additional file 2: Figure S5G). Treatment with PFT-α also promoted G1-arrested cells caused by overexpression of piR-36,712 entered to S and G2 phases (Fig. 6e and Additional file 2: Figure S5H). Furthermore, PFT-α treatment substantially increased SLUG level but decreased E-CADHERIN level (Fig. 6b), which rescued piR-36,712 overexpression-suppressed migration and invasion abilities of breast cancer cells (Fig. 6f and g, Additional file 2: Figure S5I and S5J).

We further investigated the effect of piR-36,712 in other subtypes of breast cancer cells, and found that overexpression of piR-36,712 significantly suppressed proliferation, migration and invasion of HCC1428 cells (luminal B subtype with wild type P53), whereas knockdown of piR-36,712 significantly promoted cell proliferation, migration and invasion (Additional file 2: Figure S6A-S6B and S6F-S6G). However, the tumor suppressor function of piR-36,712 cannot be observed in T47D (luminal A), BT-474 (luminal B) and MDA-MB-231 (Triple negative breast cancer, TNBC) cells, which with the mutant P53 (Additional file 2: Figure S6A and S6C-S6G). Consistent with these results, overexpression of piR-36,712 can decrease SEPW1 level and increased P53 levels in in breast cancer cells with wild type P53, but not in cells with mutant P53 (Additional file 2: Figure S6H-S6K). Together, these results indicate that the function of piR-36,712 in suppressing breast cancer cell malignant phenotypes is dependent on P53.

### piR-36,712 displays synergistic anticancer effect with chemotherapeutic agents

**Fig. 7:**
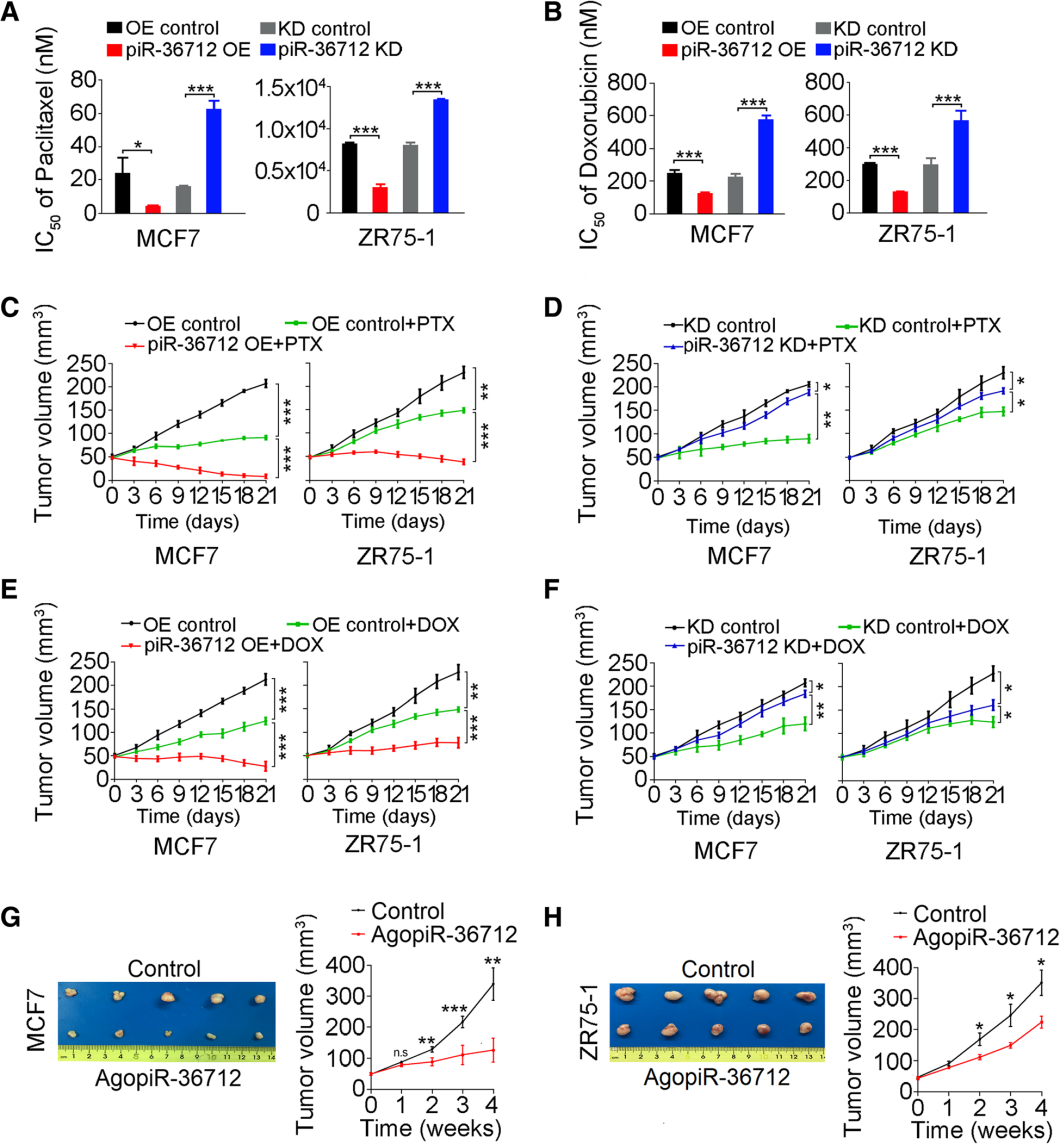
Effects of piR-36,712 on the chemosensitivity of breast cancer cells to paclitaxel (PTX) or doxorubicin (DOX). **a**, **b** Effect of overexpression (OE) or knockdown (KD) of piR-36,712 on sensitivity of MCF7 and ZR75–1 cells to PTX or DOX in vitro. **c-f** OE of piR-36,712 increased but KD of piR-36,712 decreased the sensitivity of breast cancer xenografts in mice to PTX or DOX. Shown are mean ± SEM (N = 5); *, *P* < 0.05; **, *P* < 0.01, ***, *P* < 0.001. **g**, **h** Inhibitory effect of intra-tumor administration of agopiR-36,712 on xenograft growth. Shown are pictures of tumors with or without injection of agopiR-36,712 (left panel) and the growth curves of tumors (right panel). Each point in the curves presents mean ± SEM (*N* = 5). *, *P* < 0.05; **, *P* < 0.01, ***, *P* < 0.001

Because piR-36,712 levels are correlated with PFS among patients (Fig. 1g) who were mostly received anthracycline and taxane-based adjuvant chemotherapy [40–42], we thus investigated whether piR-36,712 affects survival of breast cancer cells toward these agents. We found that overexpression of piR-36,712 in MCF7 and ZR75–1 cells significantly decreased the IC_50_ of paclitaxel or doxorubicin to these cells, but knockdown of piR-36,712 in these cells significantly increased the IC_50_ of these two agents (Fig. 7a and b, Additional file 2: Figure S7A-H). Similar effects were seen in mouse xenograft models derived from breast cancer cells with piR-36,712 overexpression or knockdown, and treated intraperitoneally with paclitaxel (15 mg/kg/week) or doxorubicin (5 mg/kg/week) for 3 weeks (Fig. 7c-f).

We further investigated whether the xenograft tumor derived from MCF7 or ZR75–1 cells can be repressed by direct administration to tumor of a chemically modified piR-36,712 mimic, agopiR-36,712. As a result, we observed that the growth of xenografts treated with agopiR-36,712 were much slower compared with those treated with scramble controls after 12 injections (Fig. 7g and h), suggesting that piR-36,712 may be an effective agent for breast cancer treatment. We also tested the effects of piR-36,712 on chemotherapy response in other subtypes of breast cancer cells, and found that the synergistically anticancer effects of piR-36,712 were reproduced in HCC1428 (wild type P53), but not in T47D, BT-474 and MDA-MB-231 cells (mutant P53) (Additional file 2: Figure S8A-S8H).

## Discussion

In this study, we have found for the first time that piR-36,712, a member of PIWI-interacting RNAs, plays a role in breast cancer as a tumor suppressor RNA and discovered a novel molecular mechanism for the functions of piRNAs. We have demonstrated that piR-36,712 can interact with RNA generated by its near-by *SEPW1P* gene, a retroprocessed pseudogene of *SEPW1*, which may decrease the expression of SEPW1 encoded by *SEPW1* though competitive interactions of their RNAs with miR-7 and miR-324. In breast cancer cells, downregulation of SEPW1 increases wild type P53, P21 and E-CADHERIN levels but decreases SLUG levels, which suppresses malignant phenotypes including proliferation, migration and invasion (Additional file 2: Figure S9). We have also shown that upregulation of piRNA-36,712 had synergistic anticancer effects with the chemotherapeutic agents for breast cancer cells. These findings shed light on a complex cellular regulation among ncRNAs, mRNAs and proteins in promoting the malignant phenotypes of cancer.

Although piRNAs were initially identified in mammalian germline, recent studies have shown that some of them are also specifically expressed in other somatic tissues and aberrantly presented in several types of human cancer [6–10]. In addition, it has been shown that certain piRNAs seem to be prognostic markers [11, 17]. The functional roles of piRNAs in human cancer are largely unknown yet. It has been suggested that certain piRNAs are involved in tumorigenesis though epigenetic mechanism such as DNA methylation [12, 43]. Other study showed that piRNA may bind to the transcriptional start site of target gene resulting in increased H3K4me3 but decreased H3K27me3 level and activation of the gene [44]. It was also reported that piR-L-163 can interact with phosphorylated ezrin-radixin-moesin proteins, resulting in accelerated DNA synthesis and G2-M cell cycle accumulation in human lung cancer cell lines [45]. Certain piRNA may also interact with TRAMP complex leading to the degradation of its targeted mRNA [46]. Here, we report for the first time a novel function for the action of piRNAs in human cancer. We have demonstrated that piR-36,712 may interact with and downregulate its neighborhood pseudogene *SEPW1P* RNA, which reduces SEPW1 protein production via a competitive mechanism between *SEPW1P* and *SEPW1* RNAs for miR-7 and miR-324. Our findings add a novel functional role to that piRNAs may play in the development and progression of human cancer.

Like other ncRNAs such as miRNAs that can interact with mRNAs inducing degradation, piRNAs also have been shown to be able to form a silencing complex and induce mRNA degradation in spermatogenesis [5, 31]. It has been documented that piRNAs may prefer to target transposable elements’ transcription but not coding genes, leading to the decreased level of their target genes in posttranscriptional level [47, 48]. In the present study, by using MS2-based RNA immunoprecipitation assays, we have provided evidence that piR-36,712 is able to directly interact with *SEPW1P* RNA but not *SEPW1* RNA. Reporter gene assays also demonstrated that piR-36,712 represses the expression of reporter gene with *SEPW1P* RNA in its 3’ UTR. The analysis of RIP with PIWIL1 antibody followed by RT-qPCR revealed a remarkably increase in recruitment of SEPW1P RNA to the piR-36,712/PIWIL1 complex in cells overexpressing piR-36,712, indicated that piR-36,712 may interact with PIWIL1 to form RISC, and mediating the degradation of SEPW1P RNA. *SEPW1P* is a retroprocessed pseudogene of *SEPW1* located in chromosome 1p34–35 12.6 Kb downstream of the piR-36,712 gene. *SEPW1P* RNA is highly homologous in sequence with mRNA of produced by *SEPW1*. Accumulating evidence has been shown that pseudogenes may also have biological function. For example, pseudogene can produce its RNA that serves as a decoy for microRNAs to relieve the repression of its protein-coding counterpart [34, 35]. In our present study, we have demonstrated that miR-7 and miR-324 can target either *SEPW1* RNA or *SEPW1P* RNA. In this context, *SEPW1P* RNA may compete with *SEPW1* RNA for these miRNAs and thus enhances SEPW1 protein expression. SEPW1 is a selenocysteine-containing protein acting as an antioxidant in vivo [49]. Recent studies have reported that SEPW1 expression is dysregulated in many types of human cancer including breast cancer [50–52]. Furthermore, SEPW1 has been involved in cell cycle process [53]. For instance, depletion of SEPW1 is able to reduce ubiquitination or facilitates phosphorylation of P53, resulting in an increased level of activated P53 and G1 cell cycle arrest [36, 37]. Previous studies revealed that P53 negatively regulate the zinc-finger protein SLUG which repressed the expression of E-CADHERIN in transcriptional level, resulting in inhibition of cancer cell invasiveness and longer metastasis-free survival of patients with cancer [38, 39, 54–57]. Our results are in line with these findings showing that P53 is negatively regulated by SEPW1 and the latter is repressed by piR-36,712 and indirectly downregulated P53 by piR-36,712 may decrease SLUG but increase E-CADHERIN level. In this context, one might expect that downregulation of piR-36,712 would increase SEPW1 protein level and thus impair P53 activity and finally enhance the malignant phenotypes of breast cancer cells with wild type P53 but not mutant P53. Since only about 20% of breast cancers carry P53 mutation [58], our results suggest that piR-36,712 may function as a tumor suppressor in most breast cancers. However, it would be interesting and important to use patients-derived cells with wild type P53 or mutant P53 to confirm these findings. In our present study, we found that piR-36,712 is significantly lower in breast tumors than in non-tumor tissues, and expression of this piRNA in tumors are negatively correlated with axillary lymph node metastasis. These results suggest that piR-36,712 may serve as a breast cancer prognostic indicator because clinical study indicated that lymph node metastasis is correlated with shorter progress-free survival time and poor outcome in breast cancer patients [59]. Also, piR-36,712 might have the potential to be used as a small nucleic acid drug to prevent or suppress axillary lymph node metastasis.

Resistance to chemotherapy is one of the hallmarks of cancer cells and the underlying mechanism has not been fully elucidated. Drug-resistance is also the main cause of cancer relapse and death of patients with cancer. P53 is a well-known tumor suppressor and also plays an important role in the reaction of cancer cells on chemotherapy [60]. Since aberrant low expression of piR-36,712 finally impaired the P53 activity and was correlated with poor PFS in our patient subjects, we explored whether piR-36,712 has the effect on survival of breast cancer cells exposed to paclitaxel and doxorubicin, two cytotoxic drugs routinely used for breast cancer treatment. As a result, we observed that piR-36,712 had a synergistic effect with these two agents both in vitro and in vivo in transplantation models. It has been shown that 7% of early breast cancer patients have local regional recurrence or axillary lymph node metastasis after radical surgery and adjuvant radiotherapy, and the portion of local relapse in triple negative breast cancer patients was up to 26% [61]. Since systemic chemotherapy had little effect in some cases, effective local therapy is thus proposed. In the present study, we also observed that direct injection of piR-36,712 analogue into transplanted tumors in mice significantly inhibited tumor growth, indicating that piR-36,712 might be an effective drug for treatment of breast cancer, especially those without P53 mutation.

## Conclusions

In the present study, we have identified piR-36,712 as a tumor suppressor that is downregulated in breast cancer and revealed the possible underlying mechanism for the action of piR-36,712 as a tumor suppressor RNA. These findings suggest that piR-36,712 might have potential clinical value in the prognostic judgment and treatment of breast cancer patients.

## Additional files

Additional file 1: Materials and Methods (DOCX 32 kb)

Additional file 2: **Figure S1**. Downregulation of piR-36,712 in breast cancer. **Figure S2**. Effects of piR-36,712 on malignant phenotypes of breast cancer cells. **Figure S3**. Effects of piR-36,712 on expressions of nearby genes and sequence alignment of SEPW1P and piR-36,712 and SEPW1. **Figure S4**. Analysis of the shared target miRNAs between SEPW1P and SEPW1. **Figure S5**. Effects of altering expression of SEPW1P, SEPW1 or P53 on oncogenic functions of piR-36,712. **Figure S6**. Ectopic piR-36,712 expression suppresses the phenotypes of breast cancer cells in a P53 dependent maner regardless molecular subtype. **Figure S7**. Ectopic piR-36,712 expression influences IC50 of paclitaxel and doxorubicin on MCF7 and ZR75–1 cells. **Figure S8**. Ectopic piR-36,712 expression influences IC50 of paclitaxel and doxorubicin on breast cancer cells in a P53 dependent maner regardless molecular subtype. **Figure S9**. Proposed acting model for the tumor suppressor role of piR-36,712 in breast cancer. (ZIP 21746 kb)

Additional file 3: **Table S1.** Baseline demographic and clinical characteristics of breast cancer patients in this study. (DOCX 35 kb)

Additional file 4: **Table S2.** Demographic and clinical characteristics of breast cancer patients recruited from Sun Yat-sen University Cancer Center (Guangzhou, *N* = 106) and Cancer Hospital, Chinese Academy of Medical Sciences (Beijing, *N* = 102). (DOCX 44 kb)

Additional file 5 : **Table S3.** Univariate and multivariate cox regression analyses for progression free survival in breast cancer patients recruited at Sun Yat-Sen University Cancer Center and Cancer Hospital, Chinese Academy of Medical Science who received radical operation (*N* = 208). (DOCX 20 kb)

Additional file 6: **Table S4.** Associations between piR-36,712 levels in tumor tissues and clinicopathological characteristics in patients with breast cancer. (DOCX 29 kb)

Additional file 7: Extended Data Sheet 1. (XLSX 72 kb)

Additional file 8: **Table S5.** Primers used for quantitative Real Time-PCR in this study. (DOCX 22 kb)

Additional file 9: **Table S6.** Sequences of synthesized probes, siRNAs and agopiR-36,712 used in this study. (DOCX 18 kb)

## Abbreviations

ER: estrogen receptor
GEO: gene expression omnibus
HER2: human epidermal growth factor receptor 2
HR: hazard ratio
piRNA: PIWI-interacting RNA
PR: progestrone receptor
SCNA: somatic copy number alteration
SEPW1: selenoprotein W
SEPW1P: selenoprotein W pseudogene 1
TCGA: the cancer genome atlas
