# Supplementary material for: PIWI-interacting RNA-36712 restrains breast cancer progression and chemoresistance by interaction with SEPW1 pseudogene SEPW1P RNA
Source: Mol Cancer. 2019 Jan 12;18:9. doi: 10.1186/s12943-019-0940-3 (PMC6330501; doi:10.1186/s12943-019-0940-3)
Supplement: Supplementary file 1 — Materials and Methods (DOCX 32 kb) [file 12943_2019_940_MOESM1_ESM.docx]

**Materials and Methods**

**Study subjects**

A total of 208 breast cancer and paired normal tissues were obtained from individuals who underwent breast resection at Sun Yat-sen University Cancer Center (SYSUCC, Guangzhou, *N* = 106) or Cancer Hospital, Chinese Academy of Medical Science (CHCAMS, Beijing, *N* = 102). All breast cancers were histopathologically diagnosed as invasive breast ductal carcinoma and tumor stage was classified according to the 7th edition of AJCC Cancer Staging System (1). The biospecimens from each individual were collected at the time of operation and immediately frozen and stored in liquid nitrogen until used. The progression-free survival time (PFS) of patients with breast cancer was measured from the date of diagnosis to the date of last follow-up or tumor progression. The follow-up information was acquired from inpatient and outpatient records and follow-up telephone calls. The last follow-up date at SYSUCC was January 1, 2017 with a median follow-up time of 63.5 months and the last follow-up date at CHCAMS was August 1, 2017 with a median follow-up time of 71.4 months. For combined sample, the median follow-up time was 65.5 months. Patients treated with adjuvant chemotherapy received the standard anthracycline-based regimen according to the NCCN Panel. The clinical characteristics and information of these patients were obtained from medical records (Supplementary Table S1 and S2). In this study, informed consent was obtained from each subject, and this study was approved by the Internal Review Boards of SYSUCC and CHCAMS.

**Public data mining and piRNA selection**

Small RNA sequencing data of 104 normal and 1103 breast cancer tissues, including 103 paired normal and cancerous samples, were obtained from TCGA database or the Cancer Genomics Hub ([see URLs](http://www.cghub.ucsc.edu/)) with the permission of the Data Access Committee. To reanalyze the TCGA breast data with a uniform approach, we recreated the raw FASTQ files based on the BAM file using BEDtools2 (v2.25.0). Then the FASTQ files were trimmed based on the criterion of “Phred quality score ≥ 20” and “reads length ≥ 21 nucleotides” to obtain high quality reads corresponding to piRNAs via FASTX-Toolkit (v0.0.13; [see URLs](http://www.cghub.ucsc.edu/)). Reads were realigned to the reference genome (hg19) using STAR with one mismatch allowed and a custom piRNA reference transcriptome using genomic coordinates for piRNA sequences obtained from the functional RNA database (v3.4) (2). Counts per million mapped reads (CPM) were calculated to measure the abundance of piRNAs using HTseq-count (v0.9.1) and R package edger (v3.18.1) (3). Only the piRNAs with CPM ≥ 1 and expressed in more than 20% of tumor samples were considered to be adequately expressed and adopted for further analyses. There were 187 piRNAs that were respectively defined as “expressed” in breast cancer. We selected the top 20 highly expressed piRNAs for investigation in the present study.

**Analysis of copy number alteration and methylation**

To dissect the somatic copy number alterations (SCNAs) and DNA methylation status of piR-36712 gene in breast cancer, we obtained the data for invasive breast carcinoma (PMID: 23000897) in TCGA database. SCNAs including homozygous deletion, heterozygous deletion, neutral, gain and amplification were determined with the GISTIC results and cutoffs (PMID: 21527027). DNA methylation probes ranging from 30 kb upstream to 10 kb downstream of the piR-36712 locus (chr1:31441013−31441043) were analyzed. Differential methylation was determined using 7 available probes including cg24722354 (chr1:31423023), cg27565803 (chr1:31423065), cg04248332 (chr1:31423071), cg00581541 (chr1:31423283), cg04078732 (chr1:31439111), cg22111442 (chr1:31441196), cg13479146 (chr1:31441314) based on Wilcoxon signed rank test.

**Cell lines and cell culture**

Human breast cancer cell lines, MCF7 and ZR75-1, and embryo kidney cell line 293T were purchased from the Cell Bank of Type Culture Collection of the Chinese Academy of Sciences, Shanghai Institute of Biochemistry and Cell Biology. MCF7 and 293T cells were maintained in DMEM medium while ZR75-1 cells were in RPMI-1640 medium supplemented with 10% fetal calf serum (Invitrogen). All cell lines were grown without antibiotics in an atmosphere of 5% CO_2_ and 99% relative humidity at 37°C. Cell lines were passaged for fewer than 6 months and were authenticated by short tandem repeat analysis. No mycoplasma infection was found for all cell lines.

**RNA isolation and quantitative real-time PCR analysis**

Total RNA of cells and tissues was extracted with TRIzol reagent (Invitrogen). First-strand cDNA was synthesized with random primer or specific miRNA or piRNA stem-loop reverse transcription primers using the Revert Aid First Stand cDNA Synthesis Kit (Thermo). The primer sequences are shown in Supplementary Table S5. RNA levels were determined by quantitative real-time PCR (qRT-PCR) in triplicate on a Roche Light Cycler 480 using the SYBR Green method (4). The RNA level was normalized against β-ACTIN or GAPDH RNA, and the piR-36712 and microRNA levels were normalized against U6 using the comparative Ct method.

**Cytoplasmic and nuclear fractionation**

Subcellular fractionation of cells was performed as described previously (5). Cytoplasmic and nuclear RNAs of MCF7 and ZR75-1 cells were isolated and purified using the Cytoplasmic & Nuclear RNA Purification Kit (Norgen Biotek).

**Northern blot assays**

Twenty µg of indicated RNA was separated on agarose gels containing formaldehyde and transferred to a Biodyne Nylon Membrane (Pall). After immobilization and pre-hybridization of RNA in DIG Easy Hybrid buffer (Roche), the membrane was hybridized overnight at 68°C in DIG Easy Hybrid buffer containing the denatured piR-36712 probe labeled with digoxigenin (synthesized by BersinBio, Supplementary Table S6). The membrane was incubated with anti-digoxigenin-AP for 30 minutes and the signal on the membrane was detected using an Odyssey infrared scanner (Li-Cor, Lincoln).

**Measurement of absolute RNA copy number per cell**

The accurate copy numbers of piR-36712, miR-7, miR-216, miR-324, miR-422, miR-641 and *SEPW1P* RNA per cell were calculated by using an absolute qRT-PCR method. We formulated standard curves with limiting dilution approaches using the corresponding synthetic RNA as standard template. Each Ct value in the curve represents an exact concentration of respective RNA. The exact copy numbers of piR-36712, miR-7, miR-216, miR-324, miR-422, miR-641 and *SEPW1P* RNA per cell were calculated based on the molecular weight and cell count.

**Lentivirus production and transduction**

To construct recombinant lentiviral vector expressing piR-36712, the precursor of piR-36712, *SNORD103C,* was inserted to the downstream of CMV promoter in pLent-Puro-GFP (Vigene Biosciences). An empty pLent-Puro-GFP vector was used as control. A short hairpin RNA (shRNA) specifically targeting piR-36712 was synthesized and subcoloned into the pSIH1-Puro-GFP lentiviral shRNA vector (Obio Technology). HEK293T cells were transfected with the vector and the lentiviral vector packaging system to produce lentivirus. A scramble shRNA served as control. MCF7 and ZR75-1 cells were infected with the corresponding concentrated lentiviruses in the presence of polybrene (Sigma-Aldrich) and were selected with puromycin to establish cell lines with stable overexpression or knockdown of piR-36712, which was verified by qRT-PCR.

**Cell viability and colony formation assays**

For cell viability assays, MCF7 (1.5 × 10^3^ per well) or ZR75-1 (2 × 10^3^ per well) cells were seeded in 96-well plates. After a certain time of cultivation, cell viability was measured with the CCK-8 Kit (Dojindo). For colony formation assays, 1,000 cells were seeded in 6-well cell culture plates and allowed to grow until visible colonies formed in complete growth medium. Cell colonies were fixed with methanol, stained with crystal violet and counted.

**In vitro invasion and migration assays**

Invasion assays were done in 24-well Millicell chamber. The 8-μm pore inserts were coated with 30 μg of matrigel (BD Biosciences). Cells (5 × 10^4^) in 200 μl of serum-free medium were added to coated filters. A 500 μl medium containing 20% fetal bovine serum was added to the lower chamber as chemo-attractant. After appropriate time in an incubator at 37°C, cells that migrated though the filter were fixed with methanol, stained with 0.5% crystal violet, and cell numbers were counted on three random fields. The migration assays were conducted in a similar fashion with cell number of 2 × 10^4^ and without coating the filters with matrigel.

**Analysis of cell cycle and apoptosis**

For cell cycle analysis, cells were collected and fixed in 70% ethanol overnight at 4°C. Single-cell suspensions were labeled with 50 μg/ml of propidium iodide (PI; Keygen Biotech) and analyzed by flow cytometry (ACEC NovoCyt). For apoptosis analysis, cells were stained with Annexin V and PI following the manufacturer’s guidelines of the Annexin V-FITC Apoptosis Detection Kit (Invitrogen). The percentage of apoptotic cells was tested by flow cytometry (ACEC NovoCyt).

**In silico analysis of RNA-miRNA binding interactions**

The miRNAs that target *SEPW1* RNA 3’untranslated region (3’ UTR) were analyzed in silico by using 4 publicly available algorithms, mirDIP, RegRNA, microCosm Targets and TargetScan (see URLs). We chose the overlapping miRNAs potentially targeting both *SEPW1* and *SEPW1P* for further experiments.

**Construction of vectors**

Reporter vectors in the psiCHECK2 backbone (Promega) were generated bearing full length of *SEPW1P* cDNA (psiCHECK2-SEPW1P) or full length of *SEPW1P* cDNA with mutations at the putative binding site of piR-36712, miR-7 or miR-324 (psiCHECK2-SEPW1P-mutpiR-36712, psiCHECK2-SEPW1P-mutmiR-7 or psiCHECK2-SEPW1P-mutmiR-324). Vectors bearing the *SEPW1* 3’ UTR (psiCHECK2-SEPW1) or the 3’ UTR with mutations at the putative binding site of miR-7 or miR-324 (psiCHECK2-SEPW1-mutmiR-7 or psiCHECK2-SEPW1-mutmiR-324) were also constructed. The full length of *SEPW1P* or *SEPW1* cDNA was subcloned into the *EcoR*I and *Xho*I sites of pcDNA3.1 vector and designated as pcDNA3.1-SEPW1P and pcDNA3.1-SEPW1, respectively. Whole genome-combined MS2-12X sequence (Obio Technology) was subcloned into the backbone of pcDNA3.1 (+) and designated as pcDNA3.1-MS2-12X. psiCHECK2-SEPW1P, psiCHECK2-SEPW1P-mutpiR-36712 and pcDNA3.1-SEPW1 were double digested and the fragments were subcloned into the *EcoR*I and *Xho*I sites of pcDNA3.1-MS2-12X and designated as pcDNA3.1-MS2-SEPW1P, pcDNA3.1-MS2-SEPW1P-mutpiR-36712 and pcDNA3.1-MS2-SEPW1, respectively. All the synthesized vectors were purchased from Obio Technology (Shanghai).

**Reporter gene assays**

MCF7 and ZR75-1 cells were seeded in 24-well plates (1 × 10^5^ cells per well) and 24 hours later, 500 ng of reporter plasmid was cotransfected with 1, 10 or 100 pmol of piR-36712, miR-7, miR-324 mimic or inhibitor (GenePharma) using lipofectamine 2000. Cells were collected 48 hours after transfection and luciferase activity was detected using the Dual-Luciferase Reporter Assay System (Promega). Renilla luciferase activity was normalized to firefly luciferase activity.

**MS2-RNA immunoprecipitation assays**

pcDNA3.1-MS2-12X, pcDNA3.1-MS2-SEPW1P, pcDNA3.1-MS2-SEPW1P-mutpiR-36712 or pcDNA3.1-MS2-SEPW1 was cotransfected with pMS2-GFP (Addgene) into MCF7 or ZR75-1 cells with lipofectamine 2000. RNA immunoprecipitation (RIP) assays were performed using GFP antibody (Roche) and the Magna RIP RNA-Binding Protein Immunoprecipitation Kit (Millipore). The immunoprecipitated RNA was subjected to qRT-PCR detection (6).

**Western blot analysis**

Protein extracts from cells were prepared using detergent-containing lysis buffer. Total protein (25 µg) was subjected to SDS-PAGE and transferred to PVDF membrane (Millipore). Antibody against SEPW1 (sc-22641, SANTA CRUZ), P53 (ab1101, Abcam), P21 (ab109520, Abcam), Slug (ab27568, Abcam), E-cadherin (#3195, Cell Signaling Technology) or β-ACTIN (60008-1-Ig, Proteintech) was used. Membrane was incubated with primary antibody overnight at 4°C and visualized with Immobilon Western Chemiluminescent HRP Substrate (Millipore).

**Pharmacological inhibition ofP53**

Cells were treated with 10 µM pifithrin (PFT)-α or DMSO as vehicle control. For functional analysis, PFT-α dissolved in DMSO was added to the culture medium. For Western blotting analysis of relative protein expressions, cells were treated with PFT-α for 48 hours before protein extraction.

**RNA decay assays**

Cells with piR-36712 overexpression or knockdown were treated with Dactinomycin. The RNA level was detected by qRT-PCR in the indicated time after Dactinomycin treatment. The relative RNA levels were normalized against GAPDH RNA using the comparative Ct method.

**Examination of chemosensitivity**

Cells were seeded in 96-well plates (4 × 10^3^ cells per well) and when adhered, a series of concentrations of paclitaxel or doxorubicin as indicated in the respective figure legends was added to the culture medium. Cell viability was measured using CCK8 Kit after incubation for 48 hours. The drug concentration corresponding to a reduction in cell survival by 50% (IC_50_) compared with that of control cells was calculated. All analyses were performed in three experiments and each had three replications.

**Animal experiments**

BALB/c nude mice, aged 3−4 weeks, were purchased from the Beijing Vital River Laboratories Animal Technology and used for the experiments after adapting to local conditions for 1 week. To examine the effects of piR-36712 on growth of implanted tumors, a total of 2 × 10^6^ of MCF7 or ZR75-1 cells with piR-36712 overexpression or knockdown were injected subcutaneously in the back flank of mice (5 per group). When a tumor was palpable, it was measured every 7 days and the volume was calculated according to the formula volume = length × width^2^ × 0.5. The experiments were ended 7 weeks after tumor implantation. To test the effects of piR-36712 on cancer cell metastasis, a total of 5 × 10^6^ luciferase labeled MCF7 or ZR75-1 cells with piR-36712 knockdown was injected via tail vein to animals (10 per group). After intraperitoneal injection of 2.0 mg luciferin (Promega) for 10 minutes, the metastases were detected using a Living Image® system (Perkin Elmer) and the quantitative data were expressed as photon flux. To examine chemosensitivity, mice (5 per group) with subcutaneous xenograft of ~50 mm^3^ were intraperitoneally injected with paclitaxel (15 mg/kg body weight/week) or doxorubicin (5 mg/kg body weight/week), respectively, for 3 weeks. Tumor volume was measured every three days. We also treated subcutaneous xenografts with agopiR-36712, a synthesized piR-36712 mimic (Ribobio, Supplementary Table S6) by intra-tumor injection to test whether piR-36712 has curative effect. Briefly, when implanted tumors in both left and right sites of mice back flanks reached a certain volume, 2 nmol of agopiR-36712 in 40 μl PBS was directly injected into tumor on the right side. Tumor on the other site was injected with agopiR-NC (Ribobio, Supplementary Table S6) as control. Treatment was delivered every other day for 28 days and tumor volume was measured once a week. Mice were sacrificed at the end of experiment and the xenografts were stripped and photographed. Experimenters were blinded to cells, drugs and solvent those were injected in the mice. All experimental procedures were performed in accordance with relevant institutional and national guidelines and regulations.

**Statistical analysis**

All statistical analyses were performed using SPSS 20.0 (IBM, US) and *P* < 0.05 was considered significant.

**URLs**

Targetscan, http://www.targetscan.org/vert_71/; miRDIP, http://ophid.utoronto.ca/mirDIP/index.jsp; microcosm Target, http://www.ebi.ac.uk/enright-srv/microcosm/cgi-bin/targets/v5/search.pl; RegRNA, http://regrna.mbc.nctu.edu.tw/index1.php; The Cancer Genomics Hub, [www.cghub.ucsc.edu/](http://www.cghub.ucsc.edu/); FASTX-Toolkit (v0.0.13), <http://hannonlab.cshl.edu/fastx_toolkit/>.

**References**

1. Sobin LH, Compton CC. TNM seventh edition: what's new, what's changed: communication from the International Union Against Cancer and the American Joint Committee on Cancer. Cancer. 2010;116 (22):5336–9

2. Mituyama T, Yamada K, Hattori E, Okida H, Ono Y, Terai G, et al. The Functional RNA Database 3.0: databases to support mining and annotation of functional RNAs. Nucleic Acids Res. 2009;37 (Database issue):D89–92

3. Robinson MD, McCarthy DJ, Smyth GK. edgeR: a Bioconductor package for differential expression analysis of digital gene expression data. Bioinformatics. 2010;26 (1):139–40

4. Schneeberger C, Speiser P, Kury F, Zeillinger R. Quantitative detection of reverse transcriptase-PCR products by means of a novel and sensitive DNA stain. PCR Methods Appl. 1995;4 (4):234–8

5. Zheng J, Huang X, Tan W, Yu D, Du Z, Chang J, et al. Pancreatic cancer risk variant in LINC00673 creates a miR-1231 binding site and interferes with PTPN11 degradation. Nat Genet. 2016;48 (7):747–57

6. Gong C, Popp MW, Maquat LE. Biochemical analysis of long non-coding RNA-containing ribonucleoprotein complexes. Methods. 2012;58 (2):88–93
