# Supplementary material for: PIWI-interacting RNA-36712 restrains breast cancer progression and chemoresistance by interaction with SEPW1 pseudogene SEPW1P RNA
Source: Mol Cancer. 2019 Jan 12;18:9. doi: 10.1186/s12943-019-0940-3 (PMC6330501; doi:10.1186/s12943-019-0940-3)
Supplement: Supplementary file 2 — Figure S1. Downregulation of piR-36,712 in breast cancer. Figure S2. Effects of piR-36,712 on malignant phenotypes of breast cancer cells. Figure S3. Effects of piR-36,712 on expressions of nearby genes and sequence alignment of SEPW1P and piR-36,712 and SEPW1. Figure S4. Analysis of the shared target miRNAs between SEPW1P and SEPW1. Figure S5. Effects of altering expression of SEPW1P, SEPW1 or P53 on oncogenic functions of piR-36,712. Figure S6. Ectopic piR-36,712 expression suppresses the phenotypes of breast cancer cells in a P53 dependent maner regardless molecular subtype. Figure S7. Ectopic piR-36,712 expression influences IC50 of paclitaxel and doxorubicin on MCF7 and ZR75–1 cells. Figure S8. Ectopic piR-36,712 expression influences IC50 of paclitaxel and doxorubicin on breast cancer cells in a P53 dependent maner regardless molecular subtype. Figure S9. Proposed acting model for the tumor suppressor role of piR-36,712 in breast cancer. (ZIP 21746 kb) [file 12943_2019_940_MOESM2_ESM.zip › 12943_2019_940_MOESM2_ESM/Supplementary Figure Legends.docx]

**SUPPLEMENTARY FIGURE LEGENDS**

**Supplementary Fig. S1 Downregulation of piR-36712 in breast cancer. (A)** Expression levels of the top 19 highly expressed piRNAs in breast cancer and paired non-tumor tissue samples from 106 individuals recruited at Sun Yat-sen University Cancer Center (Guangzhou, China), mean ± SEM; NS, no significance, *P* > 0.05. (**B** and **C**) Higher methylation status of piR-36712 gene locus in breast cancer tissues than in normal tissues based on TCGA database (*P* values based on Wilcoxon signed rank test). (**D**) Copy number alterations including homozygous deletion (Homo. Del.), heterozygous deletion (Heter. Del.), neutral, gain and amplification (Amp.) of piR-36712 gene locus in breast cancer from TCGA data.

**Supplementary Fig. S2 Effects of piR-36712 on malignant phenotypes of breast cancer cells.** (**A**) Stable overexpression (OE) and knockdown (KD) of piR-36712 in MCF7 and ZR75-1 cells (mean ± SEM; **, *P* < 0.001). (**B**) Representative images of the effect of piR-36712 expression on colony formation ability of MCF7 and ZR75-1 cells. (***C***) Representative flow cytometry images of the effect of piR-36712 expression on cell cycle progression of MCF7 and ZR75-1 cells. (**D** and **E**) Effects of piR-36712 on apoptosis of MCF7 and ZR75-1 cells. Flow cytometry images (**D**) and quantitative statistics (mean ± SEM, n=3; NS, no significance) (**E**). (**F** and **G**) Representative images of transwell assays of the effect of piR-36712 expression on the abilities of migration (**F**) and invasion (**G)** of cells. (**H** and **I**) Effects of piR-36712 expression on MCF7 cell metastasis in mice. Gross specimen of lung (**H**) and its representative HE images (**I**).

**Supplementary Fig. S3 Effects of piR-36712 on expressions of nearby genes and sequence alignment of *SEPW1P* and piR-36712 and SEPW1.** (**A**) Genomic location of piR-36712 gene and the nearby genes within about 300 kb centering piR-36712. (**B**) No effects on the expressions of 6 nearby genes by piR-36712 knockdown in MCF7 and ZR75-1 cells (mean ± SEM). KD, knockdown. (**C**) Figure shown the possible piR-36712 binding site within *SEPW1P* (*upper panel*; MEF, minimum free energy) and its homology with *SEPW1* of this region (*lower panel*). (**D**) Shown are the mutations in the putative binding sequences of *SEPW1P* and piR-36712.

**Supplementary Fig. S4** **Analysis of the shared target miRNAs between *SEPW1P* and *SEPW1*.** (**A**) Schematic of predicting the shared target miRNAs between SEPW1P and SEPW1 3’UTR by bioinformatics analysis of microcosm Target, Target SCAN, mirDIP and RegRNA. (**B**) The absolute RNA copy number measurement of indicated RNAs. (**C**) Figure shown the possible miR-7 and miR-324 binding sites within the SEPW1P and SEPW1 3’UTR. (**D**) Figure shown the site-specific mutations in the sequences of miR-7 and miR-324 target sites.

**Supplementary Fig. S5** **Effects of altering expression of *SEPW1P*, *SEPW1* or P53 on oncogenic functions of piR-36712.** (**A** and **B**) Representative flow cytometry images of the effect of overexpression **(A)** or knockdown *(****B****)* of *SEPW1P* or *SEPW1* on the cell cycle progression affected by ectopic piR-36712 expression in MCF7 and ZR75-1 cells. (**C** and **F**) Representative images of overexpression or knockdown of *SEPW1P* or *SEPW1* affecting migration (**C** and **D**) and invasion (**E** and **F**) ability induced by overexpression or knockdown of piR-36712 in MCF7 and ZR75-1 cells. (**G**) Representative images of knockdown of P53 by its pharmacologically inhibitor (PFT-α) affecting piR-36712 induced inhibition of colony formation ability of MCF7 and ZR75-1 cells. (**H**) Representative flow cytometry images of knockdown of P53 by its pharmacologically inhibitor (PFT-α) affecting cell cycle progression affected by ectopic piR-36712 expression in MCF7 and ZR75-1 cells. (**I** and **J**) Representative images of knockdown of P53 by its pharmacologically inhibitor (PFT-α) affecting migration and invasion ability induced by overexpression of piR-36712 in MCF7 and ZR75-1 cells.

**Supplementary Fig. S6** **Ectopic piR-36712 expression suppresses the phenotypes of breast cancer cells in a P53 dependent maner regardless molecular subtype.** (**A**)Effect of piR-36712 expression on HCC1428, BT-474, T47D and MDA-MB-231 cell proliferation tested by CCK8 assay (mean ± SEM; *, *P* < 0.05 and **, *P* < 0.01). (**B−E**) The fluorescent thymidine analog EdU was used to identify proliferative cells by labeling their DNA (green signal).Nuclei labeled with hoechst are in blue. Representive images (left) and quantitative statistics by flow cytometry (right) (mean ± SEM, n=3; **, *P* < 0.01 and ***, *P* < 0.001).(**F** and **G**) Effects of piR-36712 on the abilities of HCC1428, BT-474, T47D and MDA-MB-231 cell migration and invasion. Representative images (**F**) and quantitative statistics (**G**) (means ± SEM; **, *P* < 0.01; ***, *P* < 0.001).（**H−I**）Effects of piR-36712 on expressions of SEPW1 and its downstream P53 in HCC1428, BT-474, T47D and MDA-MB-231 cells.

**Supplementary Fig. S7** **Ectopic piR-36712 expression influences IC_50_ of paclitaxel and doxorubicin on MCF7 and ZR75-1 cells.** Shown are the curves of 50% inhibitory concentration of paclitaxel (PTX) and doxorubicin (DOX) on MCF7 and ZR75-1 cells with piR-36712 overexpression (OE) or knockdown (KD).

**Supplementary Fig. S8** **Ectopic piR-36712 expression influences IC_50_ of paclitaxel and doxorubicin on breast cancer cells in a P53 dependent maner regardless molecular subtype.** (**A**−**H**) Effect of overexpression (OE) or knockdown (KD) of piR-36712 on 50% inhibitory concentration of HCC1428, BT-474, T47D and MDA-MB-231 cells to PTX or DOX.

**Supplementary Fig S9** **Proposed acting model for the tumor suppressor role of piR-36712 in breast cancer.**
