# Supplementary figures and images for: PIWI-interacting RNA-36712 restrains breast cancer progression and chemoresistance by interaction with SEPW1 pseudogene SEPW1P RNA
Source: Mol Cancer. 2019 Jan 12;18:9. doi: 10.1186/s12943-019-0940-3 (PMC6330501; doi:10.1186/s12943-019-0940-3)

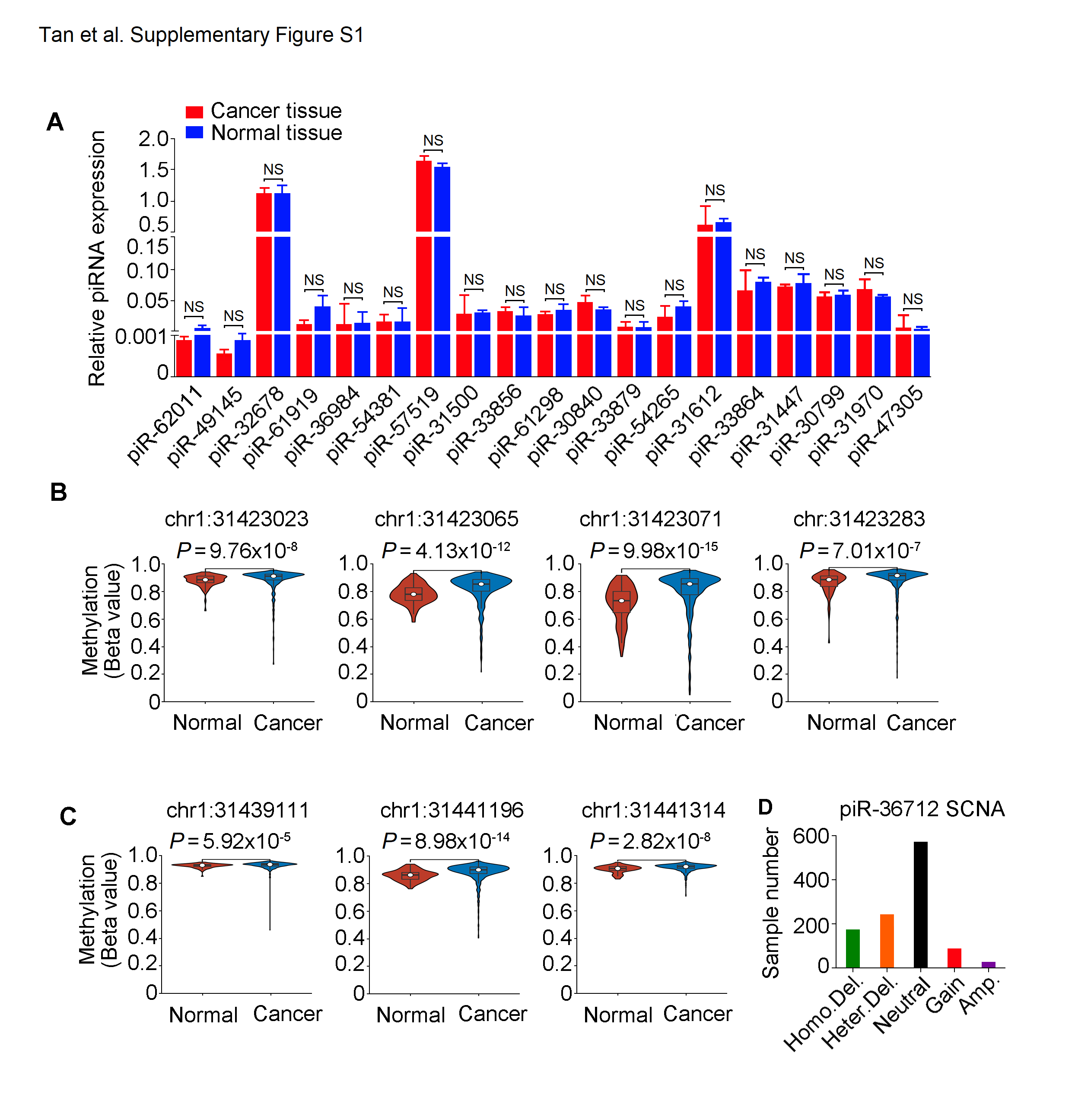

Supplement: Supplementary file 2 — Figure S1. Downregulation of piR-36,712 in breast cancer. Figure S2. Effects of piR-36,712 on malignant phenotypes of breast cancer cells. Figure S3. Effects of piR-36,712 on expressions of nearby genes and sequence alignment of SEPW1P and piR-36,712 and SEPW1. Figure S4. Analysis of the shared target miRNAs between SEPW1P and SEPW1. Figure S5. Effects of altering expression of SEPW1P, SEPW1 or P53 on oncogenic functions of piR-36,712. Figure S6. Ectopic piR-36,712 expression suppresses the phenotypes of breast cancer cells in a P53 dependent maner regardless molecular subtype. Figure S7. Ectopic piR-36,712 expression influences IC50 of paclitaxel and doxorubicin on MCF7 and ZR75–1 cells. Figure S8. Ectopic piR-36,712 expression influences IC50 of paclitaxel and doxorubicin on breast cancer cells in a P53 dependent maner regardless molecular subtype. Figure S9. Proposed acting model for the tumor suppressor role of piR-36,712 in breast cancer. (ZIP 21746 kb) [file 12943_2019_940_MOESM2_ESM.zip › 12943_2019_940_MOESM2_ESM/Supplementary Fig. S1.tif]

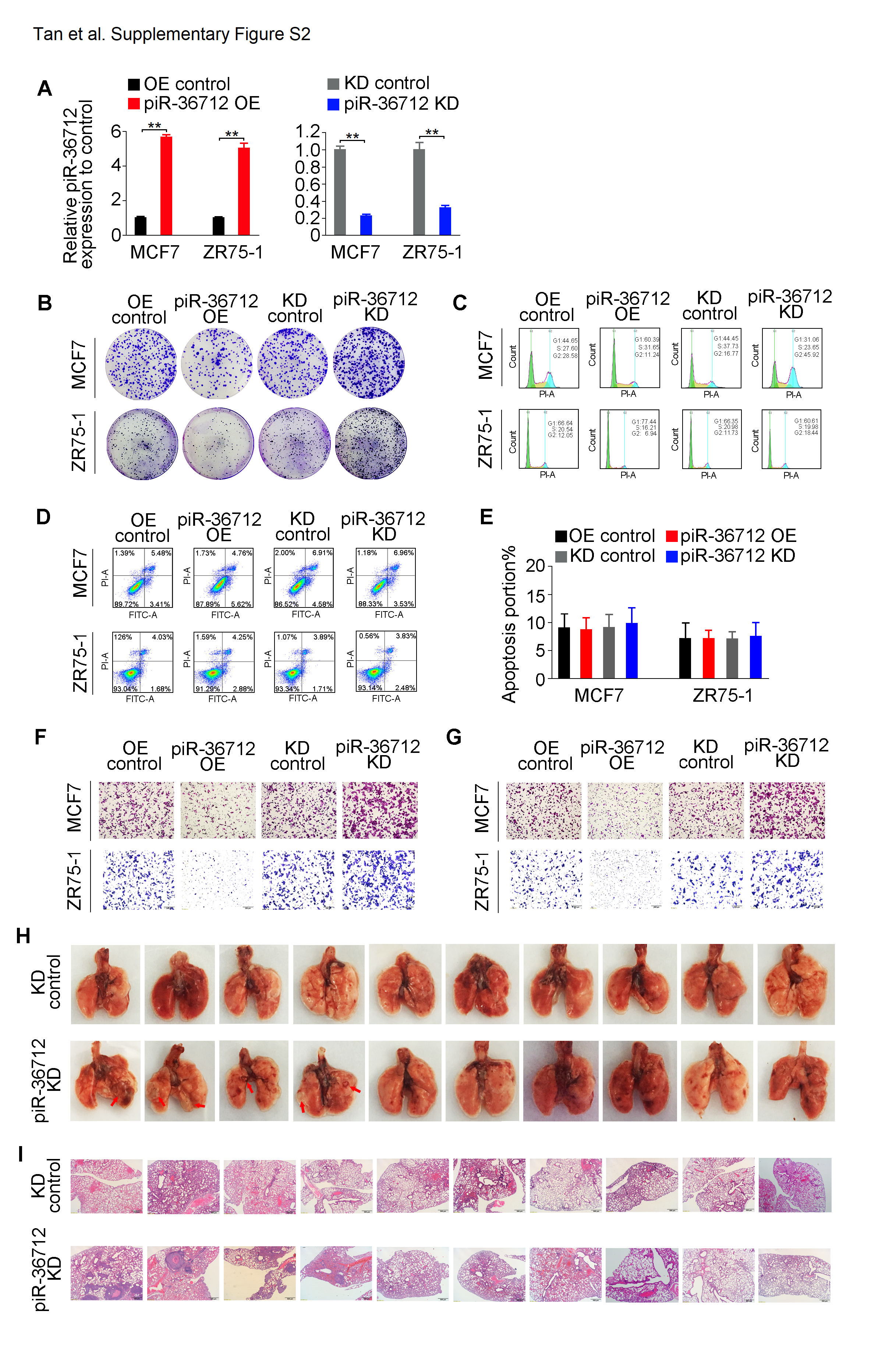

Supplement: Supplementary file 2 — Figure S1. Downregulation of piR-36,712 in breast cancer. Figure S2. Effects of piR-36,712 on malignant phenotypes of breast cancer cells. Figure S3. Effects of piR-36,712 on expressions of nearby genes and sequence alignment of SEPW1P and piR-36,712 and SEPW1. Figure S4. Analysis of the shared target miRNAs between SEPW1P and SEPW1. Figure S5. Effects of altering expression of SEPW1P, SEPW1 or P53 on oncogenic functions of piR-36,712. Figure S6. Ectopic piR-36,712 expression suppresses the phenotypes of breast cancer cells in a P53 dependent maner regardless molecular subtype. Figure S7. Ectopic piR-36,712 expression influences IC50 of paclitaxel and doxorubicin on MCF7 and ZR75–1 cells. Figure S8. Ectopic piR-36,712 expression influences IC50 of paclitaxel and doxorubicin on breast cancer cells in a P53 dependent maner regardless molecular subtype. Figure S9. Proposed acting model for the tumor suppressor role of piR-36,712 in breast cancer. (ZIP 21746 kb) [file 12943_2019_940_MOESM2_ESM.zip › 12943_2019_940_MOESM2_ESM/Supplementary Fig. S2.tif]

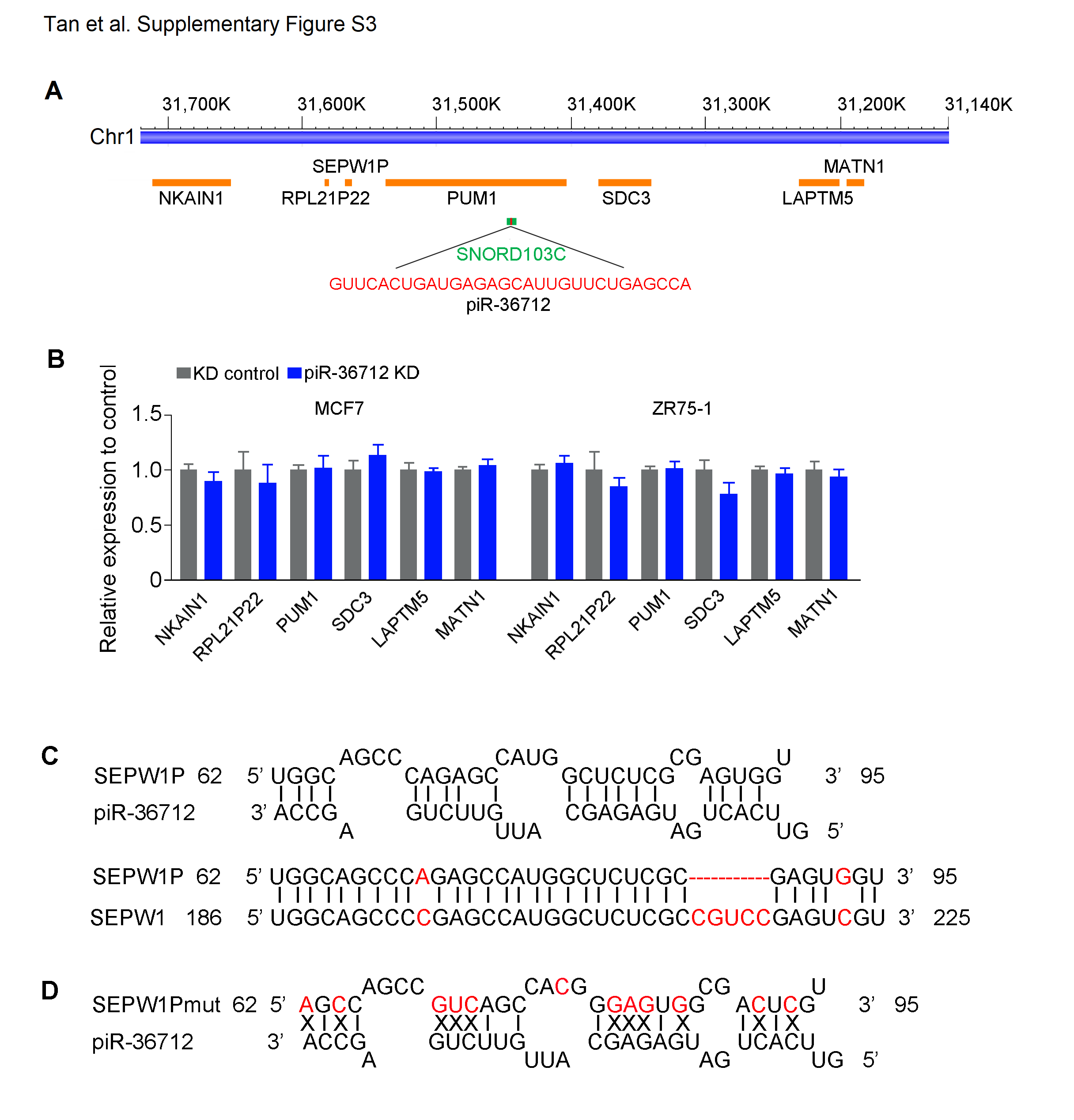

Supplement: Supplementary file 2 — Figure S1. Downregulation of piR-36,712 in breast cancer. Figure S2. Effects of piR-36,712 on malignant phenotypes of breast cancer cells. Figure S3. Effects of piR-36,712 on expressions of nearby genes and sequence alignment of SEPW1P and piR-36,712 and SEPW1. Figure S4. Analysis of the shared target miRNAs between SEPW1P and SEPW1. Figure S5. Effects of altering expression of SEPW1P, SEPW1 or P53 on oncogenic functions of piR-36,712. Figure S6. Ectopic piR-36,712 expression suppresses the phenotypes of breast cancer cells in a P53 dependent maner regardless molecular subtype. Figure S7. Ectopic piR-36,712 expression influences IC50 of paclitaxel and doxorubicin on MCF7 and ZR75–1 cells. Figure S8. Ectopic piR-36,712 expression influences IC50 of paclitaxel and doxorubicin on breast cancer cells in a P53 dependent maner regardless molecular subtype. Figure S9. Proposed acting model for the tumor suppressor role of piR-36,712 in breast cancer. (ZIP 21746 kb) [file 12943_2019_940_MOESM2_ESM.zip › 12943_2019_940_MOESM2_ESM/Supplementary Fig. S3.tif]

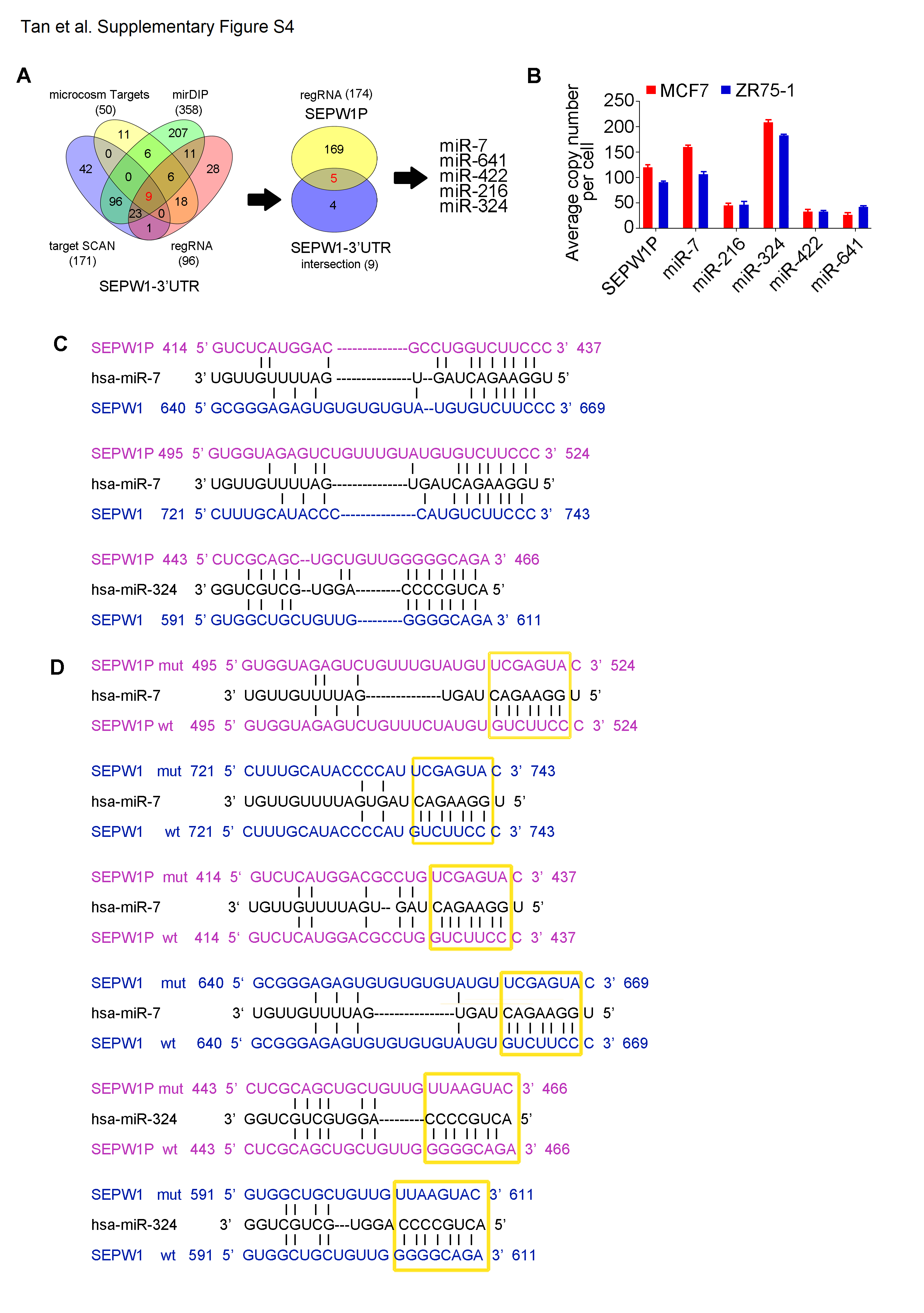

Supplement: Supplementary file 2 — Figure S1. Downregulation of piR-36,712 in breast cancer. Figure S2. Effects of piR-36,712 on malignant phenotypes of breast cancer cells. Figure S3. Effects of piR-36,712 on expressions of nearby genes and sequence alignment of SEPW1P and piR-36,712 and SEPW1. Figure S4. Analysis of the shared target miRNAs between SEPW1P and SEPW1. Figure S5. Effects of altering expression of SEPW1P, SEPW1 or P53 on oncogenic functions of piR-36,712. Figure S6. Ectopic piR-36,712 expression suppresses the phenotypes of breast cancer cells in a P53 dependent maner regardless molecular subtype. Figure S7. Ectopic piR-36,712 expression influences IC50 of paclitaxel and doxorubicin on MCF7 and ZR75–1 cells. Figure S8. Ectopic piR-36,712 expression influences IC50 of paclitaxel and doxorubicin on breast cancer cells in a P53 dependent maner regardless molecular subtype. Figure S9. Proposed acting model for the tumor suppressor role of piR-36,712 in breast cancer. (ZIP 21746 kb) [file 12943_2019_940_MOESM2_ESM.zip › 12943_2019_940_MOESM2_ESM/Supplementary Fig. S4.tif]

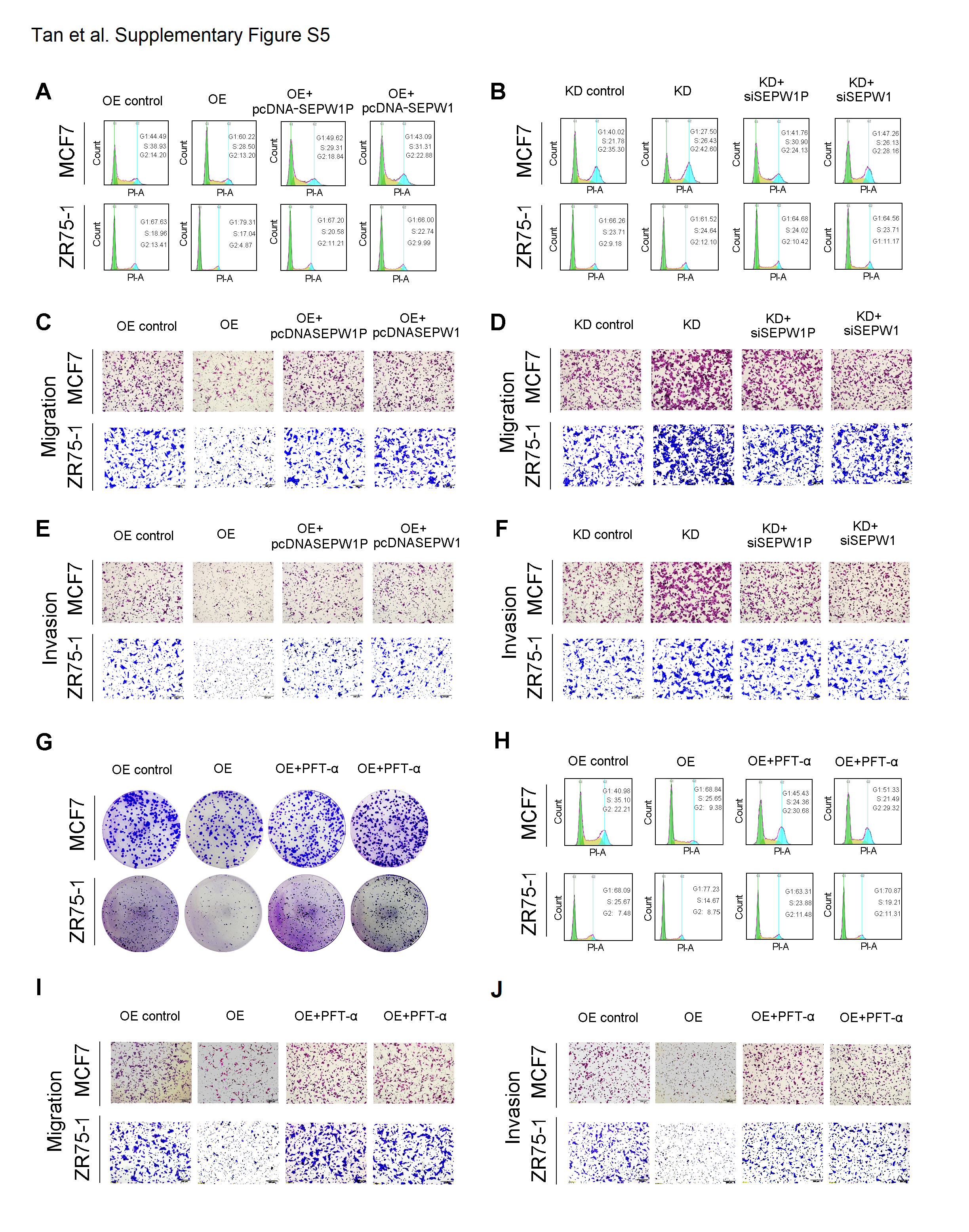

Supplement: Supplementary file 2 — Figure S1. Downregulation of piR-36,712 in breast cancer. Figure S2. Effects of piR-36,712 on malignant phenotypes of breast cancer cells. Figure S3. Effects of piR-36,712 on expressions of nearby genes and sequence alignment of SEPW1P and piR-36,712 and SEPW1. Figure S4. Analysis of the shared target miRNAs between SEPW1P and SEPW1. Figure S5. Effects of altering expression of SEPW1P, SEPW1 or P53 on oncogenic functions of piR-36,712. Figure S6. Ectopic piR-36,712 expression suppresses the phenotypes of breast cancer cells in a P53 dependent maner regardless molecular subtype. Figure S7. Ectopic piR-36,712 expression influences IC50 of paclitaxel and doxorubicin on MCF7 and ZR75–1 cells. Figure S8. Ectopic piR-36,712 expression influences IC50 of paclitaxel and doxorubicin on breast cancer cells in a P53 dependent maner regardless molecular subtype. Figure S9. Proposed acting model for the tumor suppressor role of piR-36,712 in breast cancer. (ZIP 21746 kb) [file 12943_2019_940_MOESM2_ESM.zip › 12943_2019_940_MOESM2_ESM/Supplementary Fig. S5.tif]

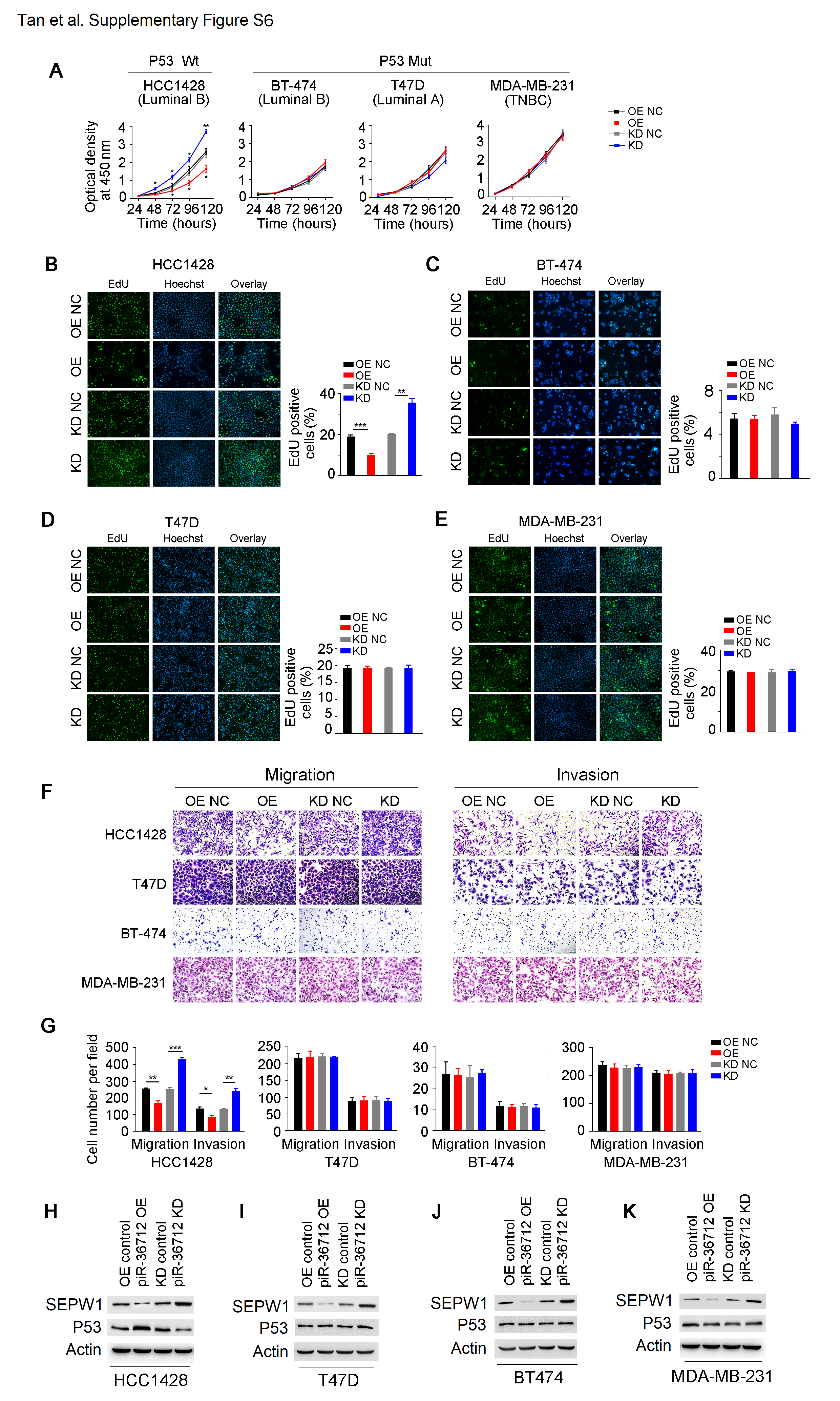

Supplement: Supplementary file 2 — Figure S1. Downregulation of piR-36,712 in breast cancer. Figure S2. Effects of piR-36,712 on malignant phenotypes of breast cancer cells. Figure S3. Effects of piR-36,712 on expressions of nearby genes and sequence alignment of SEPW1P and piR-36,712 and SEPW1. Figure S4. Analysis of the shared target miRNAs between SEPW1P and SEPW1. Figure S5. Effects of altering expression of SEPW1P, SEPW1 or P53 on oncogenic functions of piR-36,712. Figure S6. Ectopic piR-36,712 expression suppresses the phenotypes of breast cancer cells in a P53 dependent maner regardless molecular subtype. Figure S7. Ectopic piR-36,712 expression influences IC50 of paclitaxel and doxorubicin on MCF7 and ZR75–1 cells. Figure S8. Ectopic piR-36,712 expression influences IC50 of paclitaxel and doxorubicin on breast cancer cells in a P53 dependent maner regardless molecular subtype. Figure S9. Proposed acting model for the tumor suppressor role of piR-36,712 in breast cancer. (ZIP 21746 kb) [file 12943_2019_940_MOESM2_ESM.zip › 12943_2019_940_MOESM2_ESM/Supplementary Fig. S6.tif]

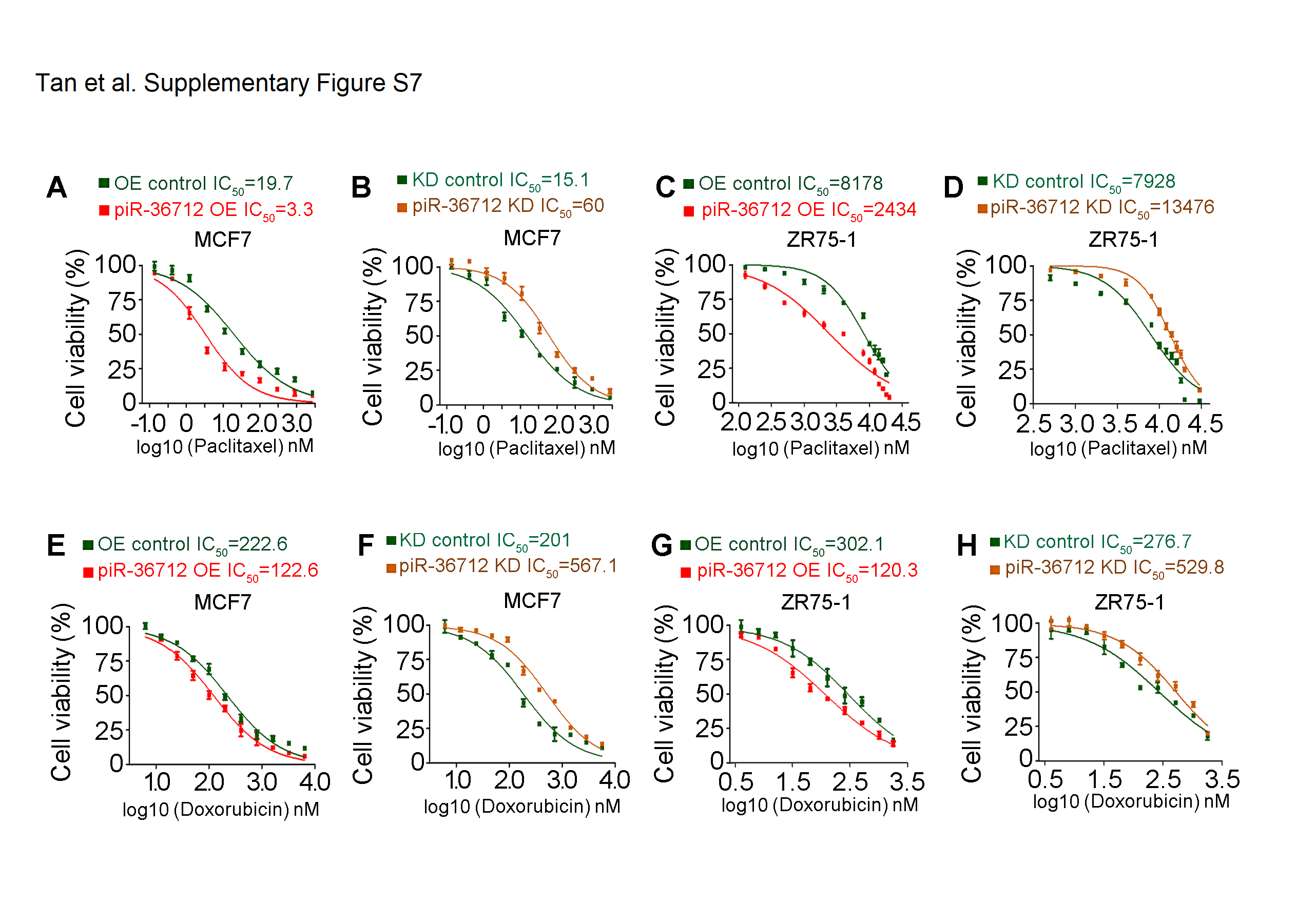

Supplement: Supplementary file 2 — Figure S1. Downregulation of piR-36,712 in breast cancer. Figure S2. Effects of piR-36,712 on malignant phenotypes of breast cancer cells. Figure S3. Effects of piR-36,712 on expressions of nearby genes and sequence alignment of SEPW1P and piR-36,712 and SEPW1. Figure S4. Analysis of the shared target miRNAs between SEPW1P and SEPW1. Figure S5. Effects of altering expression of SEPW1P, SEPW1 or P53 on oncogenic functions of piR-36,712. Figure S6. Ectopic piR-36,712 expression suppresses the phenotypes of breast cancer cells in a P53 dependent maner regardless molecular subtype. Figure S7. Ectopic piR-36,712 expression influences IC50 of paclitaxel and doxorubicin on MCF7 and ZR75–1 cells. Figure S8. Ectopic piR-36,712 expression influences IC50 of paclitaxel and doxorubicin on breast cancer cells in a P53 dependent maner regardless molecular subtype. Figure S9. Proposed acting model for the tumor suppressor role of piR-36,712 in breast cancer. (ZIP 21746 kb) [file 12943_2019_940_MOESM2_ESM.zip › 12943_2019_940_MOESM2_ESM/Supplementary Fig. S7.tif]

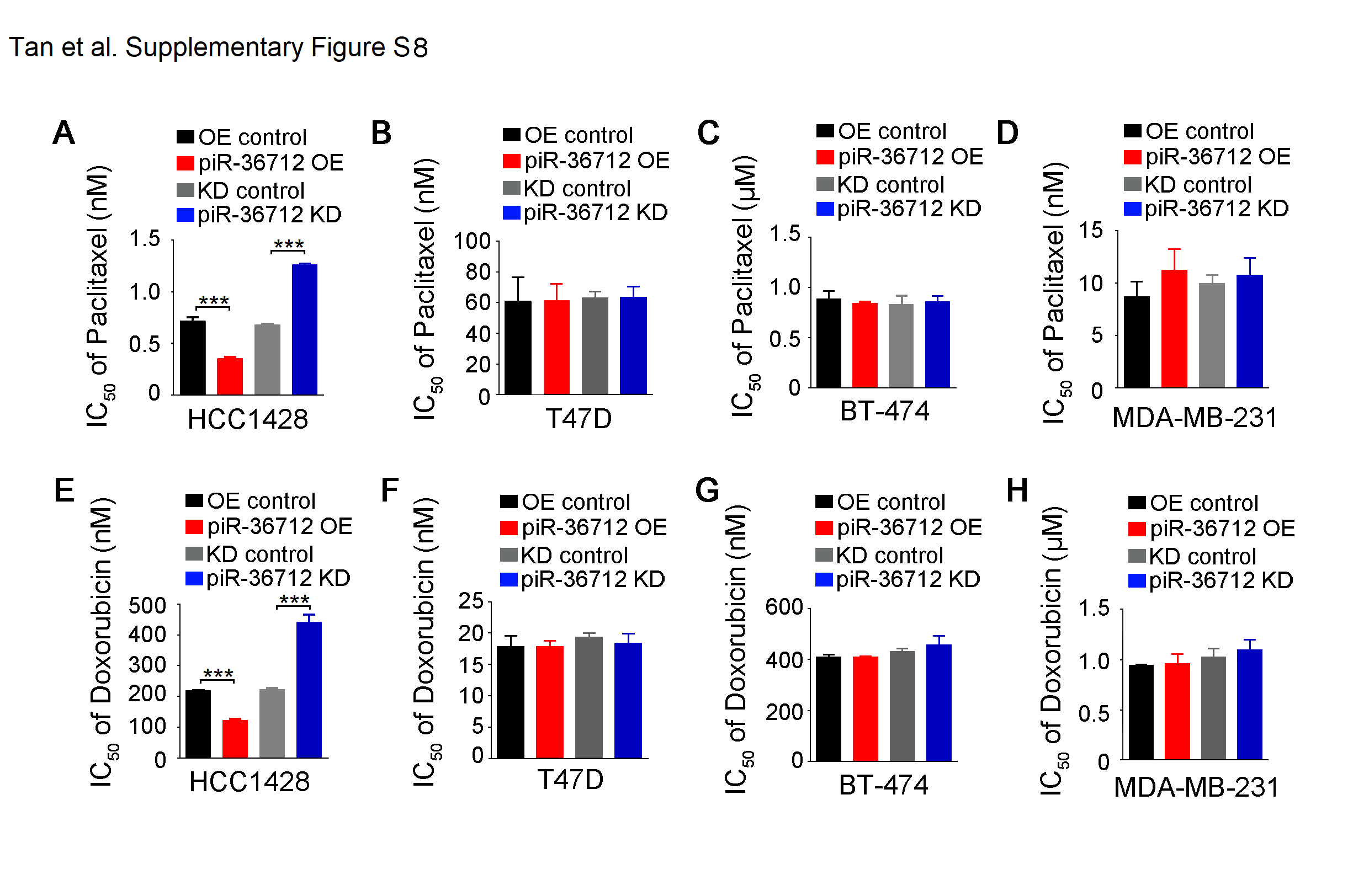

Supplement: Supplementary file 2 — Figure S1. Downregulation of piR-36,712 in breast cancer. Figure S2. Effects of piR-36,712 on malignant phenotypes of breast cancer cells. Figure S3. Effects of piR-36,712 on expressions of nearby genes and sequence alignment of SEPW1P and piR-36,712 and SEPW1. Figure S4. Analysis of the shared target miRNAs between SEPW1P and SEPW1. Figure S5. Effects of altering expression of SEPW1P, SEPW1 or P53 on oncogenic functions of piR-36,712. Figure S6. Ectopic piR-36,712 expression suppresses the phenotypes of breast cancer cells in a P53 dependent maner regardless molecular subtype. Figure S7. Ectopic piR-36,712 expression influences IC50 of paclitaxel and doxorubicin on MCF7 and ZR75–1 cells. Figure S8. Ectopic piR-36,712 expression influences IC50 of paclitaxel and doxorubicin on breast cancer cells in a P53 dependent maner regardless molecular subtype. Figure S9. Proposed acting model for the tumor suppressor role of piR-36,712 in breast cancer. (ZIP 21746 kb) [file 12943_2019_940_MOESM2_ESM.zip › 12943_2019_940_MOESM2_ESM/Supplementary Fig. S8.tif]

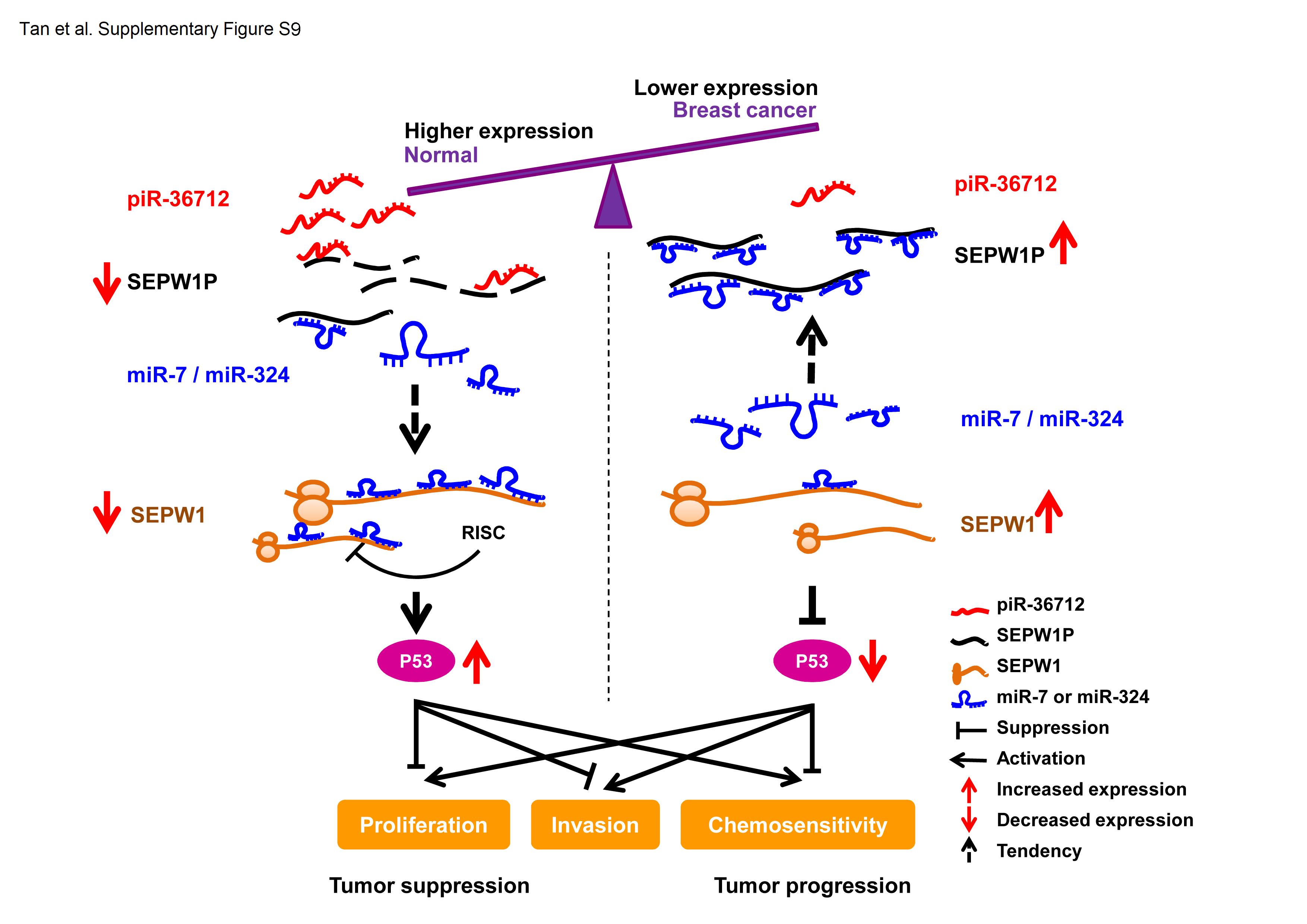

Supplement: Supplementary file 2 — Figure S1. Downregulation of piR-36,712 in breast cancer. Figure S2. Effects of piR-36,712 on malignant phenotypes of breast cancer cells. Figure S3. Effects of piR-36,712 on expressions of nearby genes and sequence alignment of SEPW1P and piR-36,712 and SEPW1. Figure S4. Analysis of the shared target miRNAs between SEPW1P and SEPW1. Figure S5. Effects of altering expression of SEPW1P, SEPW1 or P53 on oncogenic functions of piR-36,712. Figure S6. Ectopic piR-36,712 expression suppresses the phenotypes of breast cancer cells in a P53 dependent maner regardless molecular subtype. Figure S7. Ectopic piR-36,712 expression influences IC50 of paclitaxel and doxorubicin on MCF7 and ZR75–1 cells. Figure S8. Ectopic piR-36,712 expression influences IC50 of paclitaxel and doxorubicin on breast cancer cells in a P53 dependent maner regardless molecular subtype. Figure S9. Proposed acting model for the tumor suppressor role of piR-36,712 in breast cancer. (ZIP 21746 kb) [file 12943_2019_940_MOESM2_ESM.zip › 12943_2019_940_MOESM2_ESM/Supplementary Fig. S9.tif]
