# Supplementary material for: PIWI-interacting RNA-36712 restrains breast cancer progression and chemoresistance by interaction with SEPW1 pseudogene SEPW1P RNA
Source: Mol Cancer. 2019 Jan 12;18:9. doi: 10.1186/s12943-019-0940-3 (PMC6330501; doi:10.1186/s12943-019-0940-3)
Supplement: Supplementary file 3 — Table S1. Baseline demographic and clinical characteristics of breast cancer patients in this study. (DOCX 35 kb) [file 12943_2019_940_MOESM3_ESM.docx]

**Supplementary Table S1.** Baseline demographic and clinical characteristics of breast cancer patients in this study.

| Variables | Guangzhou cohort  (*N* = 106) | Beijing cohort  (*N* = 102) | Combined sample  (*N* = 208) |
| --- | --- | --- | --- |
| Age, mean (SEM) | 48.5 (0.88) | 52.9 (1.00) | 50.6 (0.68) |
| Age at diagnosis, N (%) |  |  |  |
| <50 | 58 (54.7) | 42 (41.2) | 100 (48.1) |
| ≥50 | 48 (45.3) | 60 (58.8) | 108 (51.9) |
| Menstrual status, N (%) |  |  |  |
| Post-menopause | 40 (37.7) | 54 (52.9) | 94 (45.2) |
| Pre-menopause | 66 (62.3) | 48 (47.1) | 114 (54.8) |
| Ki67%, N (%) |  |  |  |
| ≥14% | 80 (75.5) | 72 (70.6) | 152 (73.1) |
| <14% | 26 (24.5) | 30 (29.4) | 56 (26.9) |
| HER2 status, N (%) |  |  |  |
| Positive | 47 (44.3) | 37 (36.3) | 84 (40.4) |
| Negative | 59 (55.7) | 65 (63.7) | 124 (59.6) |
| PR status, N (%) |  |  |  |
| Positive | 65 (61.3) | 63 (61.8) | 128 (61.5) |
| Negative | 41 (38.7) | 39 (38.2) | 80 (38.5) |
| ER status, N (%) |  |  |  |
| Positive | 69 (65.1) | 69 (67.6) | 138 (66.3) |
| Negative | 37 (34.9) | 33 (32.4) | 70 (33.7) |
| Pathological grade |  |  |  |
| Grade I | 3 (2.8) | 4 (3.9) | 7 (3.4) |
| Grade II | 74 (69.8) | 64 (62.8) | 138 (66.3) |
| Grade III | 29 (27.4) | 34 (33.3) | 63 (30.3) |
| Number of positive node |  |  |  |
| 0 | 30 (28.3) | 40 (39.2) | 70 (33.7) |
| 1–3 | 49 (46.2) | 32 (31.4) | 81 (38.9) |
| ≥4 | 27 (25.5) | 30 (29.4) | 57 (27.4) |
| TNM stage, N (%) |  |  |  |
| Stage I | 18 (17.0) | 24 (23.5) | 42 (20.2) |
| Stage II | 56 (52.8) | 44 (43.1) | 100 (48.1) |
| Stage III | 32 (30.2) | 34 (33.4) | 66 (31.7) |
| Adjuvant chemotherapy, N (%) |  |  |  |
| Yes | 95 (89.6) | 77 (75.5) | 172 (82.7) |
| No | 11 (10.4) | 25 (24.5) | 36 (17.3) |

Guangzhou cohort refers to the patients from Sun Yat-sen University Cancer Center (Guangzhou, China); Beijing cohort refers to the patients from Cancer Hospital, Chinese Academy of Medical Sciences (Beijing, China).

Number of positive node, the number of axillary metastatic lymph node.

All the patients in our study are female and received radical operation.
