# Supplementary material for: PIWI-interacting RNA-36712 restrains breast cancer progression and chemoresistance by interaction with SEPW1 pseudogene SEPW1P RNA
Source: Mol Cancer. 2019 Jan 12;18:9. doi: 10.1186/s12943-019-0940-3 (PMC6330501; doi:10.1186/s12943-019-0940-3)
Supplement: Supplementary file 4 — Table S2. Demographic and clinical characteristics of breast cancer patients recruited from Sun Yat-sen University Cancer Center (Guangzhou, N = 106) and Cancer Hospital, Chinese Academy of Medical Sciences (Beijing, N = 102). (DOCX 44 kb) [file 12943_2019_940_MOESM4_ESM.docx]

**Supplementary Table S2**. Demographic and clinical characteristics of breast cancer patients recruited from Sun Yat-sen University Cancer Center (Guangzhou, *N* = 106) and Cancer Hospital, Chinese Academy of Medical Sciences (Beijing, *N* = 102).

| Sample ID | Age (year) | Menstrual status | Ki67% | HER2 status | PR status | ER status | Pathologic  grade | Positive node | TNMstage | Adjuvant chemotherapy | Tumor progression | PFS (month) | piR-36712 level |
| --- | --- | --- | --- | --- | --- | --- | --- | --- | --- | --- | --- | --- | --- |
| G1 | 57 | Yes | 30 | Positive | Negative | Negative | III | 22 | III | Yes | Yes | 5.2 | Low |
| G2 | 53 | Yes | 30 | Positive | Positive | Positive | III | 3 | II | Yes | Yes | 19.3 | Low |
| G3 | 46 | No | 1 | Negative | Positive | Positive | II | 0 | II | Yes | No | 73.2 | Low |
| G4 | 48 | No | 7 | Positive | Positive | Positive | II | 1 | II | Yes | No | 72.8 | Low |
| G5 | 46 | No | 70 | Negative | Negative | Negative | II | 2 | II | Yes | No | 71.2 | Low |
| G6 | 55 | Yes | 70 | Positive | Positive | Positive | I | 2 | II | Yes | Yes | 31.1 | Low |
| G7 | 44 | No | 50 | Negative | Positive | Positive | III | 0 | I | Yes | No | 66.3 | Low |
| G8 | 49 | No | 90 | Negative | Negative | Positive | II | 21 | III | Yes | Yes | 16.9 | Low |
| G9 | 47 | No | 1 | Positive | Positive | Positive | II | 2 | II | Yes | No | 71.2 | Low |
| G10 | 59 | Yes | 60 | Negative | Negative | Negative | II | 9 | III | Yes | Yes | 41.2 | Low |
| G11 | 51 | No | 70 | Negative | Positive | Positive | III | 0 | II | Yes | No | 71.2 | High |
| G12 | 31 | No | 20 | Positive | Positive | Positive | II | 1 | II | Yes | No | 69 | Low |
| G13 | 56 | Yes | 20 | Negative | Positive | Positive | II | 8 | III | Yes | Yes | 25.7 | Low |
| G14 | 69 | Yes | 20 | Negative | Negative | Positive | III | 2 | II | Yes | No | 67.3 | High |
| G15 | 45 | No | 30 | Positive | Negative | Negative | III | 1 | II | Yes | No | 70.7 | Low |
| G16 | 50 | Yes | 75 | Negative | Negative | Negative | II | 2 | II | Yes | No | 70.7 | Low |
| G17 | 54 | No | 15 | Negative | Positive | Positive | II | 5 | III | Yes | No | 70.6 | Low |
| G18 | 55 | Yes | 5 | Negative | Positive | Positive | III | 2 | III | Yes | No | 70.2 | High |
| G19 | 56 | Yes | 50 | Positive | Negative | Negative | II | 0 | II | Yes | No | 70 | High |
| G20 | 45 | Yes | 50 | Negative | Positive | Positive | II | 1 | II | Yes | No | 67.2 | High |
| G21 | 48 | No | 20 | Positive | Positive | Positive | II | 4 | III | Yes | No | 67.3 | High |
| G22 | 43 | No | 60 | Negative | Negative | Negative | III | 1 | II | Yes | No | 69.4 | High |
| G23 | 34 | No | 30 | Negative | Positive | Positive | III | 1 | II | Yes | No | 69.2 | Low |
| G24 | 41 | No | 80 | Negative | Negative | Negative | II | 2 | II | Yes | No | 69.2 | High |
| G25 | 53 | Yes | 10 | Negative | Positive | Negative | II | 10 | III | Yes | Yes | 52.8 | Low |
| G26 | 64 | Yes | 10 | Positive | Positive | Positive | II | 8 | III | No | Yes | 31.6 | Low |
| G27 | 64 | Yes | 50 | Negative | Negative | Negative | II | 0 | I | Yes | No | 69.1 | Low |
| G28 | 46 | No | 30 | Negative | Negative | Positive | II | 2 | III | Yes | Yes | 20.4 | High |
| G29 | 49 | No | 10 | Negative | Positive | Positive | II | 0 | I | Yes | No | 68.7 | High |
| G30 | 67 | No | 25 | Negative | Positive | Positive | III | 3 | II | Yes | No | 66.3 | Low |
| G31 | 41 | No | 60 | Positive | Negative | Negative | II | 1 | II | Yes | No | 67.3 | High |
| G32 | 41 | No | 30 | Negative | Positive | Positive | III | 21 | III | Yes | Yes | 41.8 | Low |
| G33 | 57 | Yes | 60 | Negative | Positive | Positive | II | 2 | II | Yes | No | 67.6 | Low |
| G34 | 53 | No | 40 | Negative | Positive | Positive | II | 13 | III | Yes | Yes | 24 | Low |
| G35 | 47 | Yes | 20 | Negative | Positive | Positive | II | 3 | II | Yes | Yes | 46.2 | Low |
| G36 | 53 | No | 5 | Negative | Positive | Positive | II | 2 | II | Yes | No | 68.1 | Low |
| G37 | 50 | No | 40 | Negative | Positive | Positive | II | 1 | II | Yes | No | 64.1 | High |
| G38 | 30 | No | 5 | Positive | Positive | Positive | II | 11 | III | Yes | Yes | 55.9 | High |
| G39 | 52 | No | 40 | Negative | Positive | Positive | III | 6 | III | No | Yes | 25.7 | Low |
| G40 | 56 | Yes | 1 | Negative | Negative | Negative | III | 1 | II | Yes | No | 67.9 | Low |
| G41 | 43 | No | 10 | Positive | Positive | Positive | III | 0 | I | No | Yes | 33.1 | Low |
| G42 | 48 | No | 20 | Negative | Positive | Positive | II | 11 | III | Yes | No | 68 | Low |
| G43 | 43 | No | 10 | Negative | Positive | Positive | III | 4 | III | Yes | No | 67.5 | High |
| G44 | 56 | Yes | 30 | Negative | Negative | Positive | II | 1 | II | Yes | No | 67.3 | Low |
| G45 | 32 | No | 30 | Positive | Negative | Positive | III | 11 | III | Yes | No | 65 | High |
| G46 | 37 | No | 1 | Negative | Positive | Positive | II | 0 | II | Yes | No | 66.3 | High |
| G47 | 35 | No | 20 | Negative | Negative | Negative | II | 0 | I | Yes | No | 66.1 | High |
| G48 | 57 | Yes | 40 | Negative | Negative | Positive | II | 22 | III | Yes | Yes | 6.2 | Low |
| G49 | 57 | Yes | 20 | Negative | Positive | Positive | II | 1 | II | Yes | Yes | 28.3 | High |
| G50 | 45 | No | 40 | Positive | Positive | Positive | II | 2 | II | Yes | Yes | 53.9 | High |
| G51 | 37 | No | 60 | Positive | Negative | Negative | II | 1 | II | Yes | No | 66.9 | Low |
| G52 | 54 | Yes | 15 | Negative | Negative | Negative | III | 3 | II | Yes | Yes | 10.1 | Low |
| G53 | 48 | No | 1 | Negative | Positive | Positive | II | 6 | III | Yes | No | 66.3 | Low |
| G54 | 39 | No | 1 | Negative | Negative | Negative | III | 9 | III | Yes | Yes | 11.9 | Low |
| G55 | 49 | No | 1 | Negative | Positive | Negative | II | 2 | III | Yes | No | 65 | High |
| G56 | 39 | No | 1 | Negative | Positive | Positive | II | 6 | III | Yes | Yes | 37.8 | Low |
| G57 | 43 | No | 30 | Negative | Positive | Positive | III | 0 | II | Yes | No | 64.1 | High |
| G58 | 49 | No | 15 | Positive | Negative | Negative | II | 2 | II | Yes | Yes | 42.1 | Low |
| G59 | 57 | Yes | 1 | Positive | Negative | Negative | II | 2 | II | Yes | No | 65.9 | High |
| G60 | 45 | No | 60 | Negative | Negative | Negative | II | 0 | II | Yes | No | 61.4 | High |
| G61 | 45 | No | 20 | Positive | Positive | Positive | II | 0 | II | Yes | No | 65.9 | High |
| G62 | 60 | Yes | 60 | Negative | Positive | Positive | II | 0 | II | Yes | No | 64.9 | High |
| G63 | 48 | No | 80 | Positive | Positive | Negative | II | 0 | II | Yes | Yes | 45.2 | High |
| G64 | 50 | No | 30 | Negative | Positive | Positive | II | 1 | II | Yes | Yes | 36.1 | High |
| G65 | 30 | No | 60 | Negative | Negative | Negative | III | 0 | II | Yes | No | 63.5 | Low |
| G66 | 32 | No | 6 | Positive | Negative | Negative | I | 15 | III | Yes | Yes | 8.9 | Low |
| G67 | 37 | No | 40 | Negative | Positive | Positive | II | 2 | I | Yes | No | 63.5 | Low |
| G68 | 30 | No | 80 | Positive | Negative | Negative | III | 4 | III | Yes | Yes | 12.1 | High |
| G69 | 52 | No | 25 | Negative | Positive | Positive | II | 0 | II | Yes | No | 65.1 | High |
| G70 | 56 | Yes | 47 | Negative | Positive | Positive | I | 12 | III | Yes | No | 64.2 | Low |
| G71 | 48 | No | 40 | Positive | Positive | Positive | II | 2 | II | Yes | No | 64.1 | High |
| G72 | 47 | No | 25 | Negative | Positive | Positive | II | 0 | II | Yes | No | 63.7 | High |
| G73 | 44 | No | 40 | Positive | Positive | Positive | II | 0 | II | Yes | No | 64.4 | High |
| G74 | 46 | No | 40 | Positive | Positive | Positive | III | 0 | II | Yes | Yes | 57.8 | High |
| G75 | 50 | No | 30 | Positive | Positive | Positive | III | 0 | II | Yes | No | 63.3 | High |
| G76 | 42 | No | 60 | Positive | Negative | Negative | II | 0 | I | Yes | No | 59.7 | Low |
| G77 | 39 | No | 70 | Negative | Positive | Negative | II | 4 | III | Yes | No | 63 | High |
| G78 | 58 | Yes | 25 | Positive | Negative | Positive | II | 2 | II | Yes | No | 63.5 | High |
| G79 | 32 | No | 20 | Positive | Positive | Positive | III | 1 | I | Yes | No | 61.7 | Low |
| G80 | 71 | Yes | 1 | Negative | Negative | Negative | II | 4 | III | Yes | No | 63.4 | High |
| G81 | 58 | Yes | 30 | Negative | Negative | Negative | III | 2 | III | Yes | Yes | 34.8 | High |
| G82 | 47 | No | 50 | Positive | Positive | Positive | II | 3 | II | Yes | Yes | 26.2 | High |
| G83 | 37 | No | 40 | Positive | Positive | Positive | II | 0 | III | Yes | No | 63 | High |
| G84 | 38 | No | 30 | Positive | Negative | Negative | II | 0 | II | Yes | No | 62.9 | High |
| G85 | 33 | No | 20 | Positive | Positive | Positive | III | 1 | II | Yes | No | 62.8 | High |
| G86 | 62 | Yes | 50 | Positive | Negative | Negative | II | 0 | II | Yes | No | 62.8 | High |
| G87 | 41 | No | 10 | Negative | Positive | Positive | II | 2 | II | Yes | No | 61.6 | Low |
| G88 | 45 | No | 10 | Negative | Positive | Positive | II | 3 | II | Yes | No | 60.8 | Low |
| G89 | 37 | No | 30 | Negative | Positive | Positive | II | 3 | II | Yes | No | 62.9 | High |
| G90 | 55 | Yes | 40 | Positive | Negative | Negative | III | 1 | II | Yes | Yes | 35.8 | Low |
| G91 | 55 | Yes | 40 | Negative | Positive | Negative | II | 0 | II | Yes | No | 61 | High |
| G92 | 59 | Yes | 30 | Positive | Positive | Positive | II | 6 | III | Yes | Yes | 8 | High |
| G93 | 40 | No | 10 | Positive | Positive | Positive | II | 5 | III | Yes | No | 61.9 | Low |
| G94 | 51 | Yes | 10 | Negative | Positive | Positive | II | 7 | III | Yes | Yes | 57.9 | Low |
| G95 | 56 | Yes | 10 | Positive | Negative | Positive | II | 2 | II | Yes | No | 61.7 | High |
| G96 | 42 | No | 30 | Positive | Negative | Negative | III | 3 | II | Yes | Yes | 3.2 | Low |
| G97 | 61 | Yes | 40 | Positive | Negative | Positive | III | 1 | I | Yes | No | 65.2 | Low |
| G98 | 54 | Yes | 20 | Positive | Negative | Positive | II | 0 | I | No | No | 67.1 | High |
| G99 | 53 | Yes | 60 | Positive | Positive | Negative | II | 1 | I | No | No | 67.3 | High |
| G100 | 55 | Yes | 5 | Positive | Negative | Positive | II | 0 | I | No | No | 60.8 | High |
| G101 | 63 | Yes | 80 | Negative | Positive | Positive | II | 1 | I | No | No | 64.7 | High |
| G102 | 58 | Yes | 40 | Positive | Negative | Positive | II | 0 | I | No | No | 69.8 | Low |
| G103 | 49 | No | 35 | Positive | Positive | Negative | II | 0 | I | No | No | 65.4 | High |
| G104 | 51 | Yes | 20 | Positive | Negative | Positive | II | 3 | I | Yes | Yes | 6.1 | Low |
| G105 | 49 | No | 25 | Positive | Positive | Negative | II | 2 | I | No | Yes | 10.3 | Low |
| G106 | 56 | Yes | 40 | Negative | Positive | Negative | II | 0 | I | No | No | 68.7 | High |
| B1 | 58 | Yes | 20 | Positive | Negative | Negative | II | 2 | II | No | No | 85.2 | Low |
| B2 | 51 | No | 80 | Negative | Positive | Negative | III | 0 | I | Yes | No | 104.7 | Low |
| B3 | 51 | Yes | 15 | Positive | Negative | Negative | II | 0 | I | Yes | No | 79.6 | Low |
| B4 | 45 | No | 60 | Negative | Negative | Negative | III | 3 | II | Yes | No | 92.2 | Low |
| B5 | 56 | No | 15 | Negative | Positive | Positive | II | 8 | III | Yes | No | 89.1 | Low |
| B6 | 58 | Yes | 15 | Negative | Positive | Positive | III | 0 | I | Yes | No | 84.8 | Low |
| B7 | 46 | Yes | 35 | Positive | Positive | Negative | II | 1 | II | Yes | No | 82.9 | Low |
| B8 | 62 | Yes | 20 | Negative | Positive | Positive | II | 1 | II | No | Yes | 15.8 | Low |
| B9 | 46 | No | 10 | Negative | Positive | Positive | II | 3 | II | Yes | No | 93.4 | Low |
| B10 | 46 | No | 30 | Negative | Negative | Positive | III | 9 | III | No | Yes | 12.3 | Low |
| B11 | 56 | Yes | 2 | Positive | Negative | Negative | III | 0 | I | No | No | 98.1 | Low |
| B12 | 43 | No | 30 | Negative | Negative | Negative | II | 1 | II | Yes | No | 71 | Low |
| B13 | 55 | Yes | 50 | Positive | Positive | Positive | II | 0 | II | Yes | No | 85.1 | High |
| B14 | 46 | No | 20 | Positive | Positive | Positive | III | 2 | II | Yes | No | 91.3 | Low |
| B15 | 50 | Yes | 40 | Positive | Negative | Positive | III | 1 | II | Yes | No | 99 | Low |
| B16 | 42 | No | 25 | Negative | Positive | Positive | II | 3 | II | Yes | No | 85.3 | Low |
| B17 | 76 | Yes | 15 | Negative | Positive | Positive | II | 0 | I | No | No | 71.3 | High |
| B18 | 59 | Yes | 40 | Positive | Positive | Positive | III | 4 | III | Yes | No | 68.8 | High |
| B19 | 70 | Yes | 10 | Negative | Positive | Positive | II | 0 | II | No | No | 73.8 | High |
| B20 | 54 | No | 20 | Positive | Negative | Negative | II | 4 | III | Yes | Yes | 12.9 | High |
| B21 | 49 | No | 10 | Negative | Positive | Positive | II | 7 | III | No | No | 84.9 | High |
| B22 | 47 | No | 10 | Negative | Positive | Positive | II | 1 | II | Yes | No | 65.6 | Low |
| B23 | 49 | No | 5 | Negative | Negative | Negative | I | 2 | II | Yes | No | 97 | Low |
| B24 | 50 | No | 50 | Negative | Positive | Positive | III | 15 | III | Yes | Yes | 21.4 | Low |
| B25 | 56 | No | 25 | Positive | Negative | Positive | II | 0 | I | No | No | 80 | High |
| B26 | 55 | Yes | 30 | Negative | Negative | Negative | III | 34 | III | Yes | Yes | 22.7 | Low |
| B27 | 50 | Yes | 90 | Positive | Positive | Positive | III | 0 | I | No | Yes | 12.5 | Low |
| B28 | 41 | No | 5 | Positive | Positive | Positive | II | 0 | I | Yes | No | 99.2 | High |
| B29 | 47 | No | 5 | Negative | Positive | Positive | II | 0 | I | Yes | No | 94.3 | Low |
| B30 | 36 | No | 15 | Negative | Positive | Positive | III | 0 | I | Yes | Yes | 24 | Low |
| B31 | 58 | Yes | 20 | Negative | Positive | Positive | II | 0 | I | Yes | No | 71.4 | High |
| B32 | 49 | No | 50 | Negative | Positive | Positive | III | 0 | II | Yes | No | 74.6 | High |
| B33 | 62 | Yes | 10 | Negative | Negative | Negative | II | 2 | II | Yes | Yes | 12 | Low |
| B34 | 68 | Yes | 30 | Positive | Negative | Positive | II | 4 | III | Yes | Yes | 53.6 | High |
| B35 | 57 | Yes | 25 | Positive | Negative | Negative | III | 4 | III | Yes | No | 59.3 | High |
| B36 | 42 | No | 30 | Positive | Negative | Negative | I | 0 | II | Yes | No | 76.6 | High |
| B37 | 35 | No | 50 | Negative | Negative | Negative | II | 12 | III | Yes | No | 115.3 | Low |
| B38 | 40 | No | 10 | Negative | Positive | Positive | II | 11 | III | Yes | No | 66.5 | High |
| B39 | 53 | Yes | 30 | Positive | Positive | Positive | II | 9 | III | No | No | 83.6 | Low |
| B40 | 52 | No | 30 | Negative | Positive | Positive | III | 6 | III | Yes | No | 79.7 | High |
| B41 | 46 | Yes | 20 | Positive | Negative | Negative | II | 2 | II | Yes | No | 91.9 | Low |
| B42 | 48 | No | 30 | Positive | Negative | Negative | II | 3 | II | Yes | No | 70.5 | Low |
| B43 | 38 | No | 10 | Negative | Positive | Positive | III | 8 | III | Yes | No | 66.6 | Low |
| B44 | 69 | Yes | 5 | Negative | Positive | Positive | II | 0 | II | Yes | No | 63.4 | High |
| B45 | 63 | Yes | 25 | Negative | Positive | Positive | II | 0 | II | Yes | No | 74.7 | High |
| B46 | 70 | Yes | 15 | Negative | Positive | Positive | II | 0 | I | No | No | 52 | High |
| B47 | 56 | Yes | 15 | Negative | Positive | Negative | II | 0 | II | Yes | No | 60.6 | High |
| B48 | 32 | No | 10 | Negative | Positive | Positive | II | 5 | III | Yes | No | 64.3 | High |
| B49 | 52 | No | 50 | Negative | Negative | Positive | II | 0 | I | Yes | Yes | 11.4 | Low |
| B50 | 60 | Yes | 20 | Negative | positive | Positive | III | 22 | III | Yes | No | 91.7 | Low |
| B51 | 77 | Yes | 3 | Positive | Negative | Negative | II | 9 | III | No | Yes | 61 | Low |
| B52 | 48 | No | 10 | Positive | Positive | Positive | II | 0 | I | Yes | No | 84.6 | Low |
| B53 | 48 | No | 5 | Negative | Positive | Positive | II | 3 | II | Yes | No | 87.3 | Low |
| B54 | 56 | No | 10 | Positive | Positive | Positive | II | 1 | II | Yes | No | 72.1 | High |
| B55 | 37 | No | 40 | Positive | Positive | Positive | II | 2 | II | Yes | Yes | 40.8 | High |
| B56 | 71 | Yes | 40 | Negative | Negative | Negative | II | 1 | II | No | Yes | 22.7 | Low |
| B57 | 36 | No | 20 | Positive | Positive | Positive | II | 0 | II | Yes | No | 74.1 | High |
| B58 | 48 | No | 20 | Negative | Positive | Positive | II | 3 | II | No | No | 76 | High |
| B59 | 55 | Yes | 20 | Negative | Positive | Positive | II | 0 | II | No | No | 58.9 | High |
| B60 | 65 | Yes | 10 | Negative | Positive | Positive | III | 2 | II | Yes | No | 69.7 | High |
| B61 | 60 | Yes | 30 | Negative | Negative | Positive | III | 0 | II | Yes | Yes | 26.6 | Low |
| B62 | 64 | Yes | 40 | Negative | Negative | Negative | III | 0 | II | Yes | No | 61.8 | High |
| B63 | 58 | Yes | 15 | Negative | Negative | Negative | III | 22 | III | Yes | Yes | 50 | Low |
| B64 | 86 | Yes | 20 | Negative | Positive | Positive | III | 3 | II | No | Yes | 22.4 | Low |
| B65 | 58 | Yes | 30 | Positive | Negative | Negative | III | 4 | III | Yes | Yes | 41.9 | High |
| B66 | 56 | Yes | 2 | Negative | Negative | Positive | II | 0 | I | Yes | No | 102.5 | Low |
| B67 | 64 | Yes | 30 | Negative | Positive | Positive | II | 3 | III | Yes | No | 103 | High |
| B68 | 38 | No | 20 | Positive | Positive | Negative | III | 0 | II | Yes | No | 65.9 | High |
| B69 | 49 | Yes | 10 | Negative | Positive | Positive | II | 2 | III | Yes | Yes | 31.4 | High |
| B70 | 55 | Yes | 10 | Negative | Positive | Positive | II | 0 | I | No | No | 75.3 | High |
| B71 | 49 | Yes | 25 | Negative | Negative | Negative | II | 5 | III | Yes | No | 59.1 | High |
| B72 | 47 | Yes | 25 | Negative | Positive | Positive | II | 0 | I | Yes | Yes | 36.2 | High |
| B73 | 65 | Yes | 35 | Negative | Positive | Positive | II | 5 | III | Yes | No | 69.6 | High |
| B74 | 75 | Yes | 10 | Negative | Positive | Positive | II | 0 | I | No | No | 67.6 | High |
| B75 | 66 | Yes | 5 | Negative | Positive | Positive | II | 0 | I | No | No | 70.7 | High |
| B76 | 40 | No | 10 | Positive | Negative | Positive | II | 5 | III | Yes | No | 65.8 | High |
| B77 | 50 | No | 50 | Positive | Negative | Negative | II | 11 | III | Yes | Yes | 35 | High |
| B78 | 56 | No | 35 | Negative | Positive | Positive | II | 0 | II | Yes | Yes | 25 | Low |
| B79 | 54 | Yes | 5 | Positive | Negative | Positive | III | 4 | III | Yes | No | 82.1 | High |
| B80 | 50 | No | 25 | Negative | Positive | Positive | II | 1 | II | Yes | No | 84.1 | High |
| B81 | 42 | No | 5 | Negative | Positive | Positive | I | 2 | II | No | No | 94.4 | Low |
| B82 | 46 | No | 20 | Positive | Positive | Negative | II | 3 | II | No | No | 61.3 | Low |
| B83 | 50 | No | 40 | Negative | Positive | Positive | II | 3 | II | Yes | Yes | 28.8 | Low |
| B84 | 49 | No | 50 | Negative | Positive | Negative | III | 9 | III | Yes | No | 78.5 | Low |
| B85 | 44 | No | 30 | Positive | Negative | Positive | II | 7 | III | Yes | Yes | 61.1 | High |
| B86 | 46 | No | 50 | Positive | Positive | Positive | III | 8 | III | Yes | No | 74.7 | Low |
| B87 | 70 | Yes | 10 | Negative | Positive | Positive | II | 0 | I | No | No | 70 | High |
| B88 | 56 | Yes | 60 | Positive | Negative | Negative | II | 43 | III | Yes | Yes | 28.1 | Low |
| B89 | 39 | No | 35 | Positive | Positive | Positive | III | 16 | III | Yes | No | 90.1 | Low |
| B90 | 53 | Yes | 30 | Negative | Positive | Positive | III | 13 | III | Yes | Yes | 33.2 | Low |
| B91 | 52 | Yes | 50 | Negative | Positive | Positive | II | 2 | II | Yes | No | 69.1 | Low |
| B92 | 48 | No | 80 | Positive | Negative | Negative | II | 0 | I | Yes | No | 79.6 | Low |
| B93 | 46 | Yes | 10 | Negative | Negative | Negative | III | 1 | I | Yes | Yes | 21.3 | Low |
| B94 | 56 | Yes | 25 | Positive | Negative | Positive | I | 0 | I | No | No | 80 | High |
| B95 | 50 | Yes | 15 | Negative | Positive | Positive | III | 2 | III | Yes | No | 72.9 | High |
| B96 | 48 | No | 20 | Positive | Negative | Negative | II | 2 | III | No | No | 83.6 | High |
| B97 | 55 | Yes | 5 | Negative | Positive | Positive | II | 3 | II | Yes | No | 79.6 | Low |
| B98 | 46 | No | 15 | Positive | Negative | Negative | III | 0 | II | Yes | No | 93.2 | High |
| B99 | 62 | Yes | 10 | Negative | Negative | Positive | III | 0 | I | No | Yes | 45.7 | High |
| B100 | 60 | Yes | 30 | Negative | Negative | Negative | II | 0 | II | Yes | No | 90.1 | High |
| B101 | 36 | No | 20 | Negative | Positive | Positive | III | 0 | II | Yes | No | 82.5 | High |
| B102 | 59 | Yes | 15 | Negative | Positive | Positive | II | 0 | II | Yes | No | 81 | High |

Menstrual status, “Yes” refers to post-menopause and “No” refers to pre-menopause.

Adjuvant chemotherapy regimens in our study were anthracycline and taxane based.

PFS, data are folLow-up time for individuals who live without progression of disease until the last folLow-up. The last folLow-up date at Sun Yat-sen University Cancer Center was 2017/01/20 with a median folLow-up time of 63.5 months, while the last folLow-up date at Cancer Hospital, Chinese Academy of Medical Sciences was 2017/8/01 with a median folLow-up time of 71.4 months. For combined sample, the median folLow-up time was 65.5 months.
