# Supplementary material for: PIWI-interacting RNA-36712 restrains breast cancer progression and chemoresistance by interaction with SEPW1 pseudogene SEPW1P RNA
Source: Mol Cancer. 2019 Jan 12;18:9. doi: 10.1186/s12943-019-0940-3 (PMC6330501; doi:10.1186/s12943-019-0940-3)
Supplement: Supplementary file 5 — : Table S3. Univariate and multivariate cox regression analyses for progression free survival in breast cancer patients recruited at Sun Yat-Sen University Cancer Center and Cancer Hospital, Chinese Academy of Medical Science who received radical operation (N = 208). (DOCX 20 kb) [file 12943_2019_940_MOESM5_ESM.docx]

**Supplementary Table S3.** Univariate and multivariate cox regression analyses for progression free survival in breast cancer patients recruited at Sun Yat-Sen University Cancer Center and Cancer Hospital, Chinese Academy of Medical Science who received radical operation (*N* = 208).

| Variable | Univariate | | |  | Multivariate | | |
| --- | --- | --- | --- | --- | --- | --- | --- |
|  | HR | 95% CI | *P* |  | HR | 95% CI | *P* |
| Age at diagnosis (<50 versus ≥50) | 0.96 | 0.48−2.28 | 0.917 |  | − | − | − |
| Menstrual status (Yes versus No) | 0.74 | 0.42−1.28 | 0.282 |  | − | − | − |
| Ki67% (<14% versus ≥14%) | 0.59 | 0.32−1.11 | 0.104 |  | − | − | − |
| HER2 status (Positive versus Negative) | 0.95 | 0.55−1.66 | 0.864 |  | − | − | − |
| PR status (Positive versus Negative) | 1.94 | 0.98−3.82 | 0.056 |  | − | − | − |
| ER status (Positive versus Negative) | 0.72 | 0.37−1.39 | 0.323 |  | − | − | − |
| Pathologic grade (I+II versus III) | 0.87 | 0.51−1.52 | 0.622 |  | − | − | − |
| Number of positive node (0 versus ≥1) | 0.62 | 0.28−1.36 | 0.229 |  | − | − | − |
| TNM stage (I+II versus III) | 0.39 | 0.22−0.70 | **0.002** |  | 0.38 | 0.23−0.62 | **<0.001** |
| Adjuvant chemotherapy (Yes versus No) | 1.33 | 0.64−2.75 | 0.448 |  | − | − | − |
| piR-36712 level (High versus Low) | 0.40 | 0.23−0.71 | **0.002** |  | 0.39 | 0.23−0.67 | **0.001** |

HR, Hazard ratio; CI, confident interval.

Adjuvant chemotherapy regimens in our study were anthracycline and taxane based.
