# Supplementary material for: PIWI-interacting RNA-36712 restrains breast cancer progression and chemoresistance by interaction with SEPW1 pseudogene SEPW1P RNA
Source: Mol Cancer. 2019 Jan 12;18:9. doi: 10.1186/s12943-019-0940-3 (PMC6330501; doi:10.1186/s12943-019-0940-3)
Supplement: Supplementary file 6 — Table S4. Associations between piR-36,712 levels in tumor tissues and clinicopathological characteristics in patients with breast cancer. (DOCX 29 kb) [file 12943_2019_940_MOESM6_ESM.docx]

**Supplementary Table S4.** Associations between piR-36712 levels in tumor tissues and clinicopathological characteristics in patients with breast cancer.

| Variable | Guangzhou cohort (N=106) | | | Beijing cohort (N=102) | | | Combined sample (N=208) | | |
| --- | --- | --- | --- | --- | --- | --- | --- | --- | --- |
|  | piR-36712 | | *P* value | piR-36712 | | *P* value | piR-36712 | | *P* value |
|  | High  (N=53) | Low  (N=53) |  | High  (N=51) | Low  (N=51) |  | High  (N=104) | Low  (N=104) |  |
| Age, mean (SEM) | 48.4(1.28) | 48.5(1.22) | 0.949 | 54.2(1.51) | 51.6(1.32) | 0.166 | 51.3(1.02) | 50.1(0.91) | 0.301 |
| Age at diagnosis, N (%) |  |  | 0.696 |  |  | 0.227 |  |  | 0.579 |
| <50 | 30(56.6) | 28(52.8) |  | 18(35.3) | 24(47.1) |  | 48(46.2) | 52(50.0) |  |
| ≥50 | 23(43.4) | 25(47.2) |  | 33(64.7) | 27(52.9) |  | 56(53.8) | 52(50.0) |  |
| Menstrual status, N (%) |  |  | 0.689 |  |  | 0.234 |  |  | 0.577 |
| Post-menopause | 19(35.8) | 21(39.6) |  | 30(58.8) | 24(47.1) |  | 49(47.1) | 45(43.3) |  |
| Pre-menopause | 34(64.2) | 32(60.4) |  | 21(41.2) | 27(52.9) |  | 55(52.9) | 59(56.7) |  |
| Ki67%, N (%) |  |  | 0.176 |  |  | 0.664 |  |  | 0.532 |
| ≥14% | 43(81.1) | 37(69.8) |  | 35(68.6) | 37(72.5) |  | 78(75.0) | 74(71.2) |  |
| <14% | 10(18.9) | 16(30.2) |  | 16(31.4) | 14(27.5) |  | 26(25.0) | 30(28.8) |  |
| HER2 status, N (%) |  |  | 0.328 |  |  | 0.537 |  |  | 0.258 |
| Positive | 26(49.1) | 21(39.6) |  | 20(39.2) | 17(33.3) |  | 46(44.2) | 38(36.5) |  |
| Negative | 27(50.9) | 32(60.4) |  | 31(60.8) | 34(66.7) |  | 58(55.8) | 66(63.5) |  |
| PR status, N (%) |  |  | 0.550 |  |  | 0.308 |  |  | 0.258 |
| Positive | 34(64.2) | 31(58.5) |  | 34(66.7) | 29(56.9) |  | 68(65.4) | 60(57.7) |  |
| Negative | 19(35.8) | 22(41.5) |  | 17(33.3) | 22(43.1) |  | 36(34.6) | 44(42.3) |  |
| ER status, N (%) |  |  | 0.839 |  |  | 0.057 |  |  | 0.240 |
| Positive | 34(64.2) | 35(66.0) |  | 39(76.5) | 30(58.8) |  | 73(70.2) | 65(62.5) |  |
| Negative | 19(35.8) | 18(34.0) |  | 12(23.5) | 21(41.2) |  | 31(29.8) | 39(37.5) |  |
| Pathological grade |  |  | 0.276 |  |  | 0.093 |  |  | 0.051 |
| Grade I+II | 41(77.4) | 36(67.9) |  | 38(74.5) | 30(58.8) |  | 79(76.0) | 66(63.5) |  |
| Grade III | 12(22.6) | 17(32.1) |  | 13(25.5) | 21(41.2) |  | 25(24.0) | 38(36.5) |  |
| No. of positive node |  |  | 0.001 |  |  | 0.005 |  |  | <0.0001 |
| 0 | 23(43.4) | 7(13.2) |  | 27(52.9) | 13(25.5) |  | 50(48.1) | 20(19.2) |  |
| ≥1 | 30(56.6) | 46(86.8) |  | 24(47.1) | 38(74.5) |  | 54(51.9) | 84(80.8) |  |
| TNM stage, N (%) |  |  | 0.204 |  |  | 0.401 |  |  | 0.766 |
| Stage I+II | 40(75.5) | 34(64.2) |  | 32(62.7) | 36(70.6) |  | 72(69.2) | 70(67.3) |  |
| Stage III | 13(24.5) | 19(35.8) |  | 19(37.3) | 15(29.4) |  | 32(30.8) | 34(32.7) |  |
| Adjuvant chemotherapy, N (%) |  |  | 0.751 |  |  | 0.491 |  |  | 0.463 |
| Yes | 47(88.7) | 48(90.6) |  | 37(72.5) | 40(78.4) |  | 84(80.8) | 88(84.6) |  |
| No | 6(11.3) | 5( 9.4) |  | 14(27.5) | 11(21.6) |  | 20(19.2) | 16(15.4) |  |

Guangzhou cohort refers to the patients from Sun Yat-sen University Cancer Center (Guangzhou, China); Beijing cohort refers to the patients from Cancer Hospital, Chinese Academy of Medical Sciences (Beijing, China).

All the patients in our study are female and received radical operation.
