# Supplementary material for: PIWI-interacting RNA-36712 restrains breast cancer progression and chemoresistance by interaction with SEPW1 pseudogene SEPW1P RNA
Source: Mol Cancer. 2019 Jan 12;18:9. doi: 10.1186/s12943-019-0940-3 (PMC6330501; doi:10.1186/s12943-019-0940-3)
Supplement: Supplementary file 8 — Table S5. Primers used for quantitative Real Time-PCR in this study. (DOCX 22 kb) [file 12943_2019_940_MOESM8_ESM.docx]

**Supplementary Table S5.** Primers used for quantitative Real Time-PCR in this study.

| Gene Symbol | Forward primer (5’→3’) | Reverse primer (5’→3’) | |
| --- | --- | --- | --- |
| *SEPW1P* | GGCTCTCGCGAGTGGTTTA | GCCGGGGGAACTCATCTTCTA | |
| *SEPW1* | TCCGAGTCGTTTATTGTGGC | CAGGGAAAGACCAGGTGTCC | |
| *NKAIN1* | TCATCTGCTTCTACTTGGAGGT | TGAGGGCTTCAATGTAGGGG | |
| *RPL21P22* | TACCTGGGTTCGACTGAAGC | CAGACATTTGGGCTGAATTGTATTT | |
| *PUM1* | CAGAACGGATTCGAGGCCA | CTCATTAATTACCTGCTGGTCTG | |
| *SDC3* | ACGCGTCCTTCCAAGAATGT | CCAGCATTGAGAGCAGGTCA | |
| *LAPTM5* | TCTCCCAGATGGGCTACCTC | GACTACGCCGATCAGTAGGC | |
| *MATN1* | GAGAGGGTCATTAGCGGACG | CCTCGGGAGATGCTGGTTTT | |
| *GAPDH* | CAAGGTCATCCATGACAACTTTG | GTCCACCACCCTGTTGCTGTAG | |
| *β-ACTIN* | CAGGGCGTGATGGTGGGCATG | GTAGAAGGTGTGGTGCCAGATT | |
| Primers used for stem-loop RT-PCR of piRNAs | | | |
| Gene Symbol | Stem-loop Reverse transcription primers (5’→3’) | Forward primer (5’→3’) | Reverse primer (5’→3’) |
| piRNA-62011* | GTCGTATCCAGTGCGTGTCGTGGAGTCGGCAATTGCACTGGATACGACACCTCACC | TTTGGCAATGGTAGAACTCACACTGG | CAGTGCGTGTCGTGGAGT |
| piRNA-49145* | GTCGTATCCAGTGCGTGTCGTGGAGTCGGCAATTGCACTGGATACGACTGTAACTC | tttTGAGGTAGTAGGTTGTATGGTTTAG | CAGTGCGTGTCGTGGAGT |
| piRNA-32678* | GTCGTATCCAGTGCGTGTCGTGGAGTCGGCAATTGCACTGGATACGACCAAGTGCG | CATTGATCATCGACACTTCGAA | CAGTGCGTGTCGTGGAGT |
| piRNA-61919* | GTCGTATCCAGTGCGTGTCGTGGAGTCGGCAATTGCACTGGATACGACTTTCAGAT | tttttTTTCTGTGTGGAATTTGAATAT | CAGTGCGTGTCGTGGAGT |
| piRNA-36984* | GTCGTATCCAGTGCGTGTCGTGGAGTCGGCAATTGCACTGGATACGACGAGGACCA | TAAAGTGCTGACAGTGCAGATAGTG | CAGTGCGTGTCGTGGAGT |
| piRNA-54381* | GTCGTATCCAGTGCGTGTCGTGGAGTCGGCAATTGCACTGGATACGACTCTCAGGT | ggggTGGATATGATGACTGATTAC | CAGTGCGTGTCGTGGAGT |
| piRNA-57519* | GTCGTATCCAGTGCGTGTCGTGGAGTCGGCAATTGCACTGGATACGACTCCTTGTA | TGGTGTATTAGTTTATACTA | CAGTGCGTGTCGTGGAGT |
| piRNA-31500* | GTCGTATCCAGTGCGTGTCGTGGAGTCGGCAATTGCACTGGATACGACAGGGTCAT | AGGTGAGCGCTTTGCGCAGTGAT | CAGTGCGTGTCGTGGAGT |
| piRNA-33856* | GTCGTATCCAGTGCGTGTCGTGGAGTCGGCAATTGCACTGGATACGACCAAAGGTG | ttCTGCAGTGATGACTTTCTTAGGA | CAGTGCGTGTCGTGGAGT |
| piRNA-61298* | GTCGTATCCAGTGCGTGTCGTGGAGTCGGCAATTGCACTGGATACGACCAAAGGTG | TTGCTGTGATGACTATCTTAGGACA | CAGTGCGTGTCGTGGAGT |
| piRNA-36712* | GTCGTATCCAGTGCGTGTCGTGGAGTCGGCAATTGCACTGGATACGACTGGCTCAG | GTTCACTGATGAGAGCATTGTTCT | CAGTGCGTGTCGTGGAGT |
| piRNA-30840* | GTCGTATCCAGTGCGTGTCGTGGAGTCGGCAATTGCACTGGATACGACGCTCAGTC | aaAGAACGTGTGGAAAACTAATGA | CAGTGCGTGTCGTGGAGT |
| piRNA-33879* | GTCGTATCCAGTGCGTGTCGTGGAGTCGGCAATTGCACTGGATACGACCAAAGGTG | CTGCGATGATGGCATTTCTTAGG | CAGTGCGTGTCGTGGAGT |
| piRNA-54265* | GTCGTATCCAGTGCGTGTCGTGGAGTCGGCAATTGCACTGGATACGACGGTCAGGC | TGGAGGTGATGAACTGTCTGA | CAGTGCGTGTCGTGGAGT |
| piRNA-31612* | GTCGTATCCAGTGCGTGTCGTGGAGTCGGCAATTGCACTGGATACGACAATCAGAA | AGTTCGTGATGGATTTGCTTTTTT | CAGTGCGTGTCGTGGAGT |
| piRNA-33864* | GTCGTATCCAGTGCGTGTCGTGGAGTCGGCAATTGCACTGGATACGACATTGTTCA | CTGCATCCACTGATAGACCTTG | CAGTGCGTGTCGTGGAGT |
| piRNA-31447* | GTCGTATCCAGTGCGTGTCGTGGAGTCGGCAATTGCACTGGATACGACGTTCAGAA | tAGGGGCTGAATGAAAATGGCCTTT | CAGTGCGTGTCGTGGAGT |
| piRNA-30799* | GTCGTATCCAGTGCGTGTCGTGGAGTCGGCAATTGCACTGGATACGACAATCAGAA | ACTGTGTGCTGATTGTCACG | CAGTGCGTGTCGTGGAGT |
| piRNA-31970* | GTCGTATCCAGTGCGTGTCGTGGAGTCGGCAATTGCACTGGATACGACCATCAGAC | CAAAGTGATTGGTACCTCGTT | CAGTGCGTGTCGTGGAGT |
| piRNA-47305* | GTCGTATCCAGTGCGTGTCGTGGAGTCGGCAATTGCACTGGATACGACTAAGGGGT | TGAATCTGACAACAGAGGCTTACGAC | CAGTGCGTGTCGTGGAGT |
| miR-7* | GTCGTATCCAGTGCGTGTCGTGGAGTCGGCAATTGCACTGGATACGACACAACAAAA | TCACCGGGTGTAAATCAGCTTGGTCGTA | CAGTGCGTGTCGTGGAGT |
| miR-324* | GTCGTATCCAGTGCGTGTCGTGGAGTCGGCAATTGCACTGGATACGACCCAGCAG | TACCCGTAATCTTCATAATCCGAGGTCG | CAGTGCGTGTCGTGGAGT |
| miR-216* | GTCGTATCCAGTGCGTGTCGTGGAGTCGGCAATTGCACTGGATACGACTCACATT | ggggAAATCTCTGCAGGC | CAGTGCGTGTCGTGGAGT |
| miR-422* | GTCGTATCCAGTGCGTGTCGTGGAGTCGGCAATTGCACTGGATACGACGCCTTCTGA | ggggttACTGGACTTAGGGT | CAGTGCGTGTCGTGGAGT |
| miR-641* | GTCGTATCCAGTGCGTGTCGTGGAGTCGGCAATTGCACTGGATACGACGAGGTGAC | AAAGACATAGGATAGAGT | CAGTGCGTGTCGTGGAGT |
| U6 | AACGCTTCACGAATTTGCGT | CTCGCTTCGGCAGCACA | AACGCTTCACGAATTTGCGT |

*Homo sapiens origin*.*
