# Supplementary material for: PIWI-interacting RNA-36712 restrains breast cancer progression and chemoresistance by interaction with SEPW1 pseudogene SEPW1P RNA
Source: Mol Cancer. 2019 Jan 12;18:9. doi: 10.1186/s12943-019-0940-3 (PMC6330501; doi:10.1186/s12943-019-0940-3)
Supplement: Supplementary file 9 — Table S6. Sequences of synthesized probes, siRNAs and agopiR-36,712 used in this study. (DOCX 18 kb) [file 12943_2019_940_MOESM9_ESM.docx]

Supplementary Table S6**.** Sequences of synthesized probes, siRNAs and agopiR-36712 used in this study

| Probe used for Northern blot assay | |
| --- | --- |
| piRNA-36712 | 5’DIG-11-dUTP-TGGCTCAGAACAATGCTCTCATCAGTGAAC-3’ |
| Sequences of small interfering RNAs | |
| Scramble | 5’-UUCUCCGAACGUGUCACGUTT-3’ |
| SEPW1-siRNA | 5’-CAUGAUAGGAAGGACUGAATT-3’ |
| SEPW1P-siRNA | 5’-CCGGGUUCUUUGACGUAAUTT-3’ |
| AgopiR-36712 used for in vivo assay | |
| AgomiR-NC22 | 5'chol-UUUGUACUACACAAAAGUACUG(2'OMe)-3' |
| AgopiR-36712 | 5'chol-GUUCACUGAUGAGAGCAUUGUUCUGAGCCA(2'OMe)-3' |
